# The WNT Inhibitor PRI-724 Depletes Endogenous SOX2 and Blocks SOX2 Upregulation by Therapeutic Regimes

**DOI:** 10.3390/cancers18213569

**Published:** 2026-11-06

**Authors:** Alexander R. Sage, Daisy V. Gonzalez, Briana D. Ormsbee Golden, Ethan Metz, Rebecca E. Oberley-Deegan, Peter Xie, Donald W. Coulter, Chittalsinh Raulji, Grinu Mathew, Angie Rizzino

**Affiliations:** 1 Eppley Institute for Research in Cancer and Allied Diseases, University of Nebraska Medical Center, Omaha, NE 68198, USApxie@unmc.edu (P.X.);; 2 Department of Biochemistry and Molecular Biology, University of Nebraska Medical Center, Omaha, NE 68198, USA; 3 Fred & Pamela Buffett Cancer Center, University of Nebraska Medical Center, Omaha, NE 68198, USA; 4 Department of Pediatrics, Hematology and Oncology Division, University of Nebraska Medical Center, Omaha, NE 68198, USAchittalsinh.raulji@unmc.edu (C.R.); 5 Child Health Research Institute, University of Nebraska Medical Center, Omaha, NE 68198, USA

**Keywords:** SOX2, WNT inhibitors, PRI-724, vemurafenib, gemcitabine, radiation, cancer, transcriptome

## Abstract

**Background/Objectives:** The stem cell transcription factor SOX2 is expressed in more than 20 types of cancer. SOX2 expression is also elevated by a wide range of clinically used drugs and has been linked causally to drug and radiation resistance. Hence, SOX2 is recognized as an important therapeutic target. This study set out to identify a small molecule inhibitor that reduces SOX2 in a wide range of tumor types. **Methods:** In this study, the WNT inhibitor, PRI-724 (Foscenvivint), was tested for its ability to downregulate SOX2 in ten tumor cell lines using western blot analysis. We also used western blot analysis to examine whether PRI-724 blocks the upregulation of SOX2 by clinically used drugs and radiation. Additionally, we examined the overall effects of PRI-724 on the transcriptome by performing RNA-Seq and STRING analysis. **Results:** PRI-724 not only downregulated SOX2 in ten different tumor cell lines representing eight different tumor types, but also blocked the upregulation of SOX2 induced by vemurafenib, gemcitabine and radiation. Besides downregulating SOX2 at the protein and RNA level, without altering the stability of SOX2 RNA, PRI-724 significantly modified the transcriptome. STRING analysis of the upregulated and downregulated genes indicates that PRI-724 altered the expression of genes involved in stress-response pathways, including endoplasmic reticulum (ER) stress. **Conclusions:** The WNT inhibitor PRI-724 downregulates SOX2 expression in a wide range of tumor cell types and blocks the upregulation of SOX2 by clinically used regimens. PRI-724 also modulates the expression of genes associated with stress-response pathways.

## Simple Summary

The stem cell transcription factor SOX2 is expressed in over twenty types of human cancer and is linked directly to drug and radiation resistance. As such, SOX2 is a significant therapeutic target. In this study, we report that the WNT inhibitor PRI-724 (Foscenvivint) decreases the expression of SOX2 in ten different human tumor cell lines representing eight types of cancer. Equally significant, PRI-724 blocks the upregulation of SOX2 by three standard-of-care clinical treatments: vemurafenib (Zelboraf), gemcitabine (Gemzar) and radiation. In addition to reducing the expression of SOX2, we report that PRI-724 significantly modifies gene expression globally at the RNA level, including genes involved in stress response pathways.

## 1. Introduction

The stem cell transcription factor SOX2 is a member of the SOX (SRY-related HMG-box) family of transcription factors. The defining feature of SOX2 is its essential roles during embryogenesis and later stages of mammalian development [1]. SOX2 is also one of the core four Yamanaka reprogramming factors [2]. Beyond its essential roles during mammalian development, SOX2 has been implicated in a broad range of human malignancies. Since 2006, SOX2 has been shown to be expressed in over 20 types of human cancer, including pancreatic ductal adenocarcinoma, medulloblastoma, glioblastoma, diffuse midline gliomas, melanoma, prostate cancer, lung cancer, and clear cell ovarian cancer [3]. By virtue of its ability to bind to a large network of genes and its pioneering activity, SOX2 promotes a broad constellation of malignant properties, including altered proliferation, epithelial–mesenchymal transition, invasion, metastasis, and resistance to numerous cancer therapies [3].

A growing body of evidence indicates that SOX2 plays critical roles in the function and maintenance of the cancer stem cell populations of multiple tumor types [3]. This vital subpopulation of tumor cells is responsible for the self-renewal of tumor-initiating cells of the tumor and differentiation into the bulk of the tumor cell population. In several different tumors, SOX2 has been shown to be elevated in their infrequently proliferating cancer stem cell populations [4,5,6]. Importantly, several lines of evidence indicate that high levels of SOX2 play an instructive role in limiting the proliferation of cancer stem cells [7], as they do in normal stem cells during mammalian development [8,9]. In the case of cancer, elevation of SOX2 from an inducible promoter in over a dozen human tumor cell lines has been shown to inhibit tumor cell growth, both in vitro and in vivo [10,11,12,13,14]. When SOX2 was elevated in vivo in three different tumor types, it was found to induce a reversible state of tumor growth arrest until SOX2 was returned to basal endogenous levels [13]. These findings, along with SOX2 depletion studies showing reduced tumor growth [7], demonstrate that SOX2 dosage carefully controls tumor growth.

SOX2 has also been implicated directly in resistance to drugs used clinically for the treatment of cancer [3]. This includes resistance of lung and ovarian cancer to platinum-based chemotherapy, and resistance of breast cancer to tamoxifen [15,16,17]. Moreover, clinically used drugs have been shown to increase SOX2 expression. This includes gemcitabine in pancreatic ductal adenocarcinoma cells, enzalutamide in prostate tumor cells, and vemurafenib in melanoma cells [18,19,20]. Equally important, knocking down SOX2 has been shown to restore responses to drugs used in preclinical models [19]. Like drug resistance, SOX2 expression has been implicated directly in resistance to radiotherapy, and radiation has been shown to increase SOX2 expression in several cancers, including non-small cell lung cancer, pancreatic cancer, colorectal cancer, glioblastoma, and H3K27M diffuse midline gliomas [21,22,23,24,25,26]. In the case of non-small cell lung cancer cells, elevating SOX2 reduces their radiosensitivity, while knocking down SOX2 increases their radiosensitivity [22].

It is widely believed that depletion of SOX2 could offer significant clinical benefit. However, targeting SOX2 itself at the protein level has proven very difficult, because SOX2, like other transcription factors, has been found to be largely undruggable [27]. With the exception of the DNA-binding domain of SOX2, the remainder of the protein is largely unstructured, and targeting the DNA-binding domain of SOX2 is likely to lead to significant off-target effects, because the DNA-binding domains of SOX family members are highly conserved [28]. Rather than targeting SOX2 itself, there are multiple reports of small-molecule inhibitors and herbal supplements that reduce the expression of SOX2 in cultured cells. These include napabucasin (BBI608), pevonedistat (MLN4924), spiropyazoline oxindole, triptolide, salinomycin, resveratrol, curcumin, and epigallocatechin-3-gallate (EGCG from green tea) [29,30,31,32,33,34,35,36]. However, most of these inhibitors have been shown to reduce SOX2 expression in only one or two tumor types.

Given the challenges of targeting SOX2, the work in this report initially set out to identify a small molecule inhibitor that reduces endogenous SOX2 expression in a broad range of human tumor cell types. For this purpose, we focused on WNT signaling. Besides the prominent roles of WNT signaling in stem cell fate decisions [37], WNT signaling has been implicated in several SOX2-positive cancers [38,39,40]. Moreover, the first-generation β-catenin/CBP inhibitor, ICG-001, has been reported to decrease SOX2/CD44-positive tumor sphere formation by nasopharyngeal carcinoma cells [40] and to downregulate SOX2 in cervical tumor cells [39]. In this study, we tested two different WNT inhibitors, including the second-generation β-catenin/CBP inhibitor, PRI-724 (Foscenvivint). PRI-724 is an analog of ICG-001 with improved pharmacokinetic properties that has been tested in clinical trials [41]. Here we show that PRI-724 not only reduces SOX2 expression in ten different tumor cell lines, but it also blocks the upregulation of SOX2 by three different clinically used cancer therapies. Additionally, we determined that PRI-724 exerts broad effects on the transcriptome in brain tumor cells.

## 2. Materials and Methods

### 2.1. Cell Culture

ONS76 and UW228 cells (cellosaurus.org accessions CVCL_1624 and CVCL_8585, respectively) and their SOX2 inducible counterparts (i-SOX2-ONS76 and i-SOX2-UW228) have been described previously [13,14], as have T3M4 and L3.6 cells [11] (cellosaurus.org accessions CVCL_4056 and CVCL_0384, respectively). A-549 cells and A-375 cells were obtained from ATCC (CRM-CCL-185 and CRL-1619, respectively, ATCC, Manassas, VA, USA). Bin-67 cells were obtained from Jessica Long (UW-Madison, Madison, WI, USA; cellosaurus.org accession CVCL_S987). U373-MG (Uppsala) cells and SF8628 cells were obtained from Sigma (08061901 and SCC127, respectively, SIGMA, St. Louis, MO, USA). CWR-R1ca cells were obtained from Sigma (SCC118, SIGMA). All cells, except CWR-R1ca cells, were cultured in Dulbecco’s Modified Eagle Medium (12100046, Thermo Fisher Scientific, Waltham, MA, USA) supplemented with 10% HyClone Characterized Fetal Bovine Serum (SH30910.03, Cytiva, Thermo Fisher Scientific) and 1% penicillin-streptomycin (P3032-10MU & S9137-25G, SIGMA). CWR-R1ca cells were cultured in RPMI-1640 medium (R8758, SIGMA) supplemented with 10% fetal bovine serum and 1% penicillin-streptomycin.

The following inhibitors from Selleck Chemicals (Houston, TX, USA) were dissolved and diluted in dimethyl sulfoxide (DMSO; D2438, SIGMA): PRI-724 (Foscenvivint, S8968), LGK-974 (WNT974, S7143), and PLX4032 (Vemurafenib, S1267). Gemcitabine (Gemcitabine HCl, S1149, Selleck Chem, Houston, TX, USA) was dissolved and diluted in sterile filtered water. Treatment of cells was conducted by replenishing cell-adhered plates with premixed medium containing the desired concentration of each inhibitor. Control cell plates for all experiments were replenished with premixed medium containing the appropriate volume of solvent for the molecules used. In all studies where DMSO was included, it was used at 0.2% final concentration. Where PRI-724 was used at a single concentration, 1 μM was selected as the standard concentration used across different cell lines, because that was the lowest concentration that provided the maximal downregulation of SOX2 protein in those cell lines. Cell irradiation was conducted using an RS2000 (RadSourceTechnologies, Inc., Buford, GA 30518, USA) with single-dose radiation at 2 Gray (Gy) per minute with varying exposure time to achieve the indicated Gy, and experiments were concluded with cell harvest 16 h after radiation exposure. Cell lines not obtained from the American Type Culture Collection were verified by genetic analysis performed by the UNMC Molecular Diagnostic Laboratory. All cell lines tested negative for the presence of mycoplasma.

### 2.2. Western Blotting

Harvested cells were lysed using RIPA buffer, which was supplemented with protease and phosphatase inhibitors as previously described [42]. Western blot analysis was performed as previously described [42]. Each separate set of protein samples was loaded in 4–20% Tris-Glycine gels (XP04202BOX, Thermo Fisher Scientific), transferred to methanol-activated PVDF membranes (IPVH20200, SIGMA), and processed for protein detection with ECF substrate (45000947, Fisher Scientific), followed by densitometry measurements as described previously [42]. The following antibodies from Cell Signaling Technology (CST, Danvers, MA, USA) were used for western blot analysis: SOX2 (#3579, 1:1000), β-Tubulin (#2146, 1:1000), and AP-linked anti-rabbit IgG secondary antibody (#7054, 1:5000). An additional SOX2 antibody from CST (#23064, 1:1000) as well as an additional AP-linked anti-rabbit IgG secondary antibody from Sigma (A3687, 1:5000, SIGMA) was also used for some experiments.

### 2.3. Proliferation and Cell Cycle Analysis

Cell proliferation was assessed by direct cell counting using a Z1 Coulter^®^ Particle Counter (Beckman Coulter, Inc., Indianapolis, IN, USA). Triplicate cell counts were obtained for ONS76 cells seeded in 6-well plates and treated the next day with either DMSO only as a control or with increasing PRI-724 treatments: 0.1 µM, 0.25 µM, and 0.5 µM for 72 h. DMSO was at a concentration of 0.1% for all wells. Cells were then released from the wells with 500 µL Trypsin, stopped with 500 µL of complete media, mixed well with pipetting to ensure a single-cell suspension, and then 500 µL of each cell suspension was added to a separate cup of 9.5 mL Isoton II Diluent (C96980, Beckman Coulter, Inc.) to count on the Coulter counter. Changes in cell proliferation after PRI-724 treatment were confirmed by MTT assays performed in triplicate, as described previously [42]. For cell cycle analysis, i-SOX2-ONS76 cells were plated at subconfluent densities and cultured in the presence of either DMSO or 1 µM PRI-724. Cells were then stained for cell cycle analysis according to the Telford method, as described previously [14]. Floating cells were collected and included in all cell cycle analyses. Flow cytometry analysis of triplicate samples was performed by the UNMC Flow Cytometry Research Facility. Statistical significance was determined using a two-tailed Student’s *t*-test, with a significance threshold of *p* < 0.05.

### 2.4. RNA Isolation, cDNA Synthesis, and RT-qPCR

For RNA studies, cells were seeded in 60 mm plates at 0.5 × 10^6^ cells per plate and given one day to attach before the medium was changed to medium premixed with either DMSO or 1 µM PRI-724. For the time course study, RNA was isolated at the specified treatment time points. For the actinomycin D experiment, plates were treated with DMSO or 1 µM PRI-724 for 16 h before each plate was given 20 µg of actinomycin D (A1410, Sigma) and then harvested for RNA at 0, 1, 2.5, and 5 h time points. RNA isolation was performed using the RNeasy kit (74104, Qiagen, Germantown, MD, USA), with cell lysis conducted directly in the plates using RLT buffer and vortexing after transferring the samples to appropriately labeled 1.5 mL tubes, as outlined in the manufacturer’s protocols. RNA was then purified using RNeasy columns and eluted in 35 µL RNase-free water. RNA was measured on a NanoDrop 2000 (Thermo Fisher Scientific). cDNA was synthesized using 1 µg of RNA and the Agilent High Fidelity 1st Strand cDNA synthesis kit (Agilent Technologies, La Jolla, CA, USA), as described in detail previously [42]. Primers used in the RT-qPCR are provided in Table 1. RT-qPCR amplification and quantification were performed in triplicate as described previously [42], with *GAPDH* used as a loading control and the Applied Biosystems QuantStudio 3 Real-Time PCR System (Thermo Fisher Scientific) used for the RT-qPCR reactions.

### 2.5. Colony Assay and Staining

Cells were seeded in T-25 flasks at 500 cells per flask. The cells were allowed to attach for 24 h. After this initial 24 h period, the medium of the “Control–DMSO” flask was changed to fresh medium premixed with DMSO, while the medium of the remaining flasks was changed to fresh medium premixed with 1 µM PRI-724. The flasks were then incubated for 72 h. After this 72 h incubation period, the conditions of two “PRI-724 Removed” flasks were changed to not contain PRI-724. To that end, all four flasks had their respective medium removed and were washed with 2 mL of 1X PBS. The “Control–DMSO” and the two “PRI-724 Removed” flasks were then replenished with fresh medium premixed with DMSO, while the “PRI-724 Sustained” flask was replenished with fresh medium premixed with 1 µM PRI-724. All flasks were cultured for another 72 h. After this 72 h period and every 72 h thereafter, medium was removed from all flasks, followed by a PBS wash, and replenishment with fresh medium premixed with DMSO (“Control–DMSO” and both “PRI-724 Removed” flasks) or premixed with 1 µM PRI-724 (“PRI-724 Sustained” flask). Staining of the control flasks was performed before colonies could grow into each other, and the total growth time was noted. All other flasks were stained 72 h after the control flasks were stained unless otherwise specified.

For staining, the medium was removed from the plate, and the plate was washed with 1X PBS. After the removal of the PBS wash, each plate was given 2 mL of 200 proof ethanol for 15 min at room temperature. Then the fixative agent was removed, and each plate was gently washed with 3 mL of 1X PBS, which was removed as well, before adding 3 mL of 0.1% Crystal Violet to each plate. The Crystal Violet was allowed to incubate on the cells for 20 min before it was removed by gentle rinses with water until no free Crystal Violet Dye remained within the flask. Each flask was then allowed to dry before the colonies were counted and photos were taken using an Olympus IMT-2 microscope (Artisan Technology Group ®, Champaign, IL, USA) at 10X magnification and a Canon EOS Rebel T6 camera (Canon Inc., Ota-ku, Tokyo, Japan) with a Diagnostic Instruments Microscope PA1-10A Camera Adapter (Munday Scientific Instruments, Sanford, NC, USA).

### 2.6. mRNA Sequencing

RNA was isolated as described above from ONS76, UW228, and SF8628 cell lines cultured as biological triplicates with DMSO only or 1 µM PRI-724 for 5 h. RNA sequencing was performed by Novogene (Sacramento, CA, USA). Novogene assessed the purity and quality of the RNA samples and performed library preparation and transcriptome sequencing on an Illumina NovaSeq^TM^ X Plus platform (Illumina, Inc., San Diego, CA, USA). Paired-end reads were aligned to the human genome version hg38 using HISAT2 [43]. FeatureCounts was used to count the number of reads mapped to each gene, and then the FPKM of each gene was calculated based on the length of the gene and the number of reads mapped to the gene. Differential gene expression analysis was assessed using DESeq2 [44] (version 1.52.0), and significantly changed genes were required to have a Benjamini–Hochberg adjusted *p*-value of <0.05 and a Log2FoldChange of ≥0.5 or ≤−0.5.

### 2.7. STRING Analysis

Genes altered by treatment with PRI-724 were first sorted for padj < 0.05 from the list of genes in the RNA-seq data for each cell line. Genes found to have a Log2FC of >0.5 were considered upregulated genes, and genes found to have a Log2FC < −0.5 were considered downregulated genes. After applying these filters to the RNA-seq data for all cell lines, the lists of gene names were then separated based on whether they were either upregulated or downregulated before being uploaded to separate Venny 2.1 [45] Venn diagrams. A list of commonly altered genes amongst all three cell lines was retrieved from the upregulated and downregulated genes in the Venn diagrams. The lists of commonly altered genes were then combined and used to generate the STRING [46] map. The STRING map parameters were set to a minimum interaction score of 0.7 (High confidence), and the search was conducted using “Homo sapiens” under the organism selection screen. Then MCL clustering was performed with an inflation parameter of 1.1, after which the unconnected nodes and gene clusters were removed from the original list before a new STRING map was created with a new MCL clustering inflation parameter set to 1.4, following the same 0.7 confidence score and organism selection. Cluster descriptions were determined from the top Gene Ontology Biological Processes for each individual cluster under the Analysis tab provided by the STRING program. Version 12.0 was used.

## 3. Results

### 3.1. Targeting SOX2 Expression via WNT Pathway Inhibitors in Multiple Cell Lines

To explore whether WNT inhibitors downregulate SOX2, we initially tested the effects of two WNT inhibitors, PRI-724 and LGK-974, on the expression of SOX2. PRI-724 is a downstream WNT inhibitor that is reported to block the interaction between β-Catenin and CBP [40] while LGK-974 is an upstream WNT inhibitor that interferes with WNT signaling by inhibiting the O-acyltransferase activity of PORCN and the release of WNT ligands [47]. For these initial studies, we tested the effects of the two WNT inhibitors on the ONS76 (sonic hedgehog medulloblastoma) cell line, because of the critical role of SOX2 in the cancer stem cell population of this medulloblastoma subtype [48].

**Figure 1:**
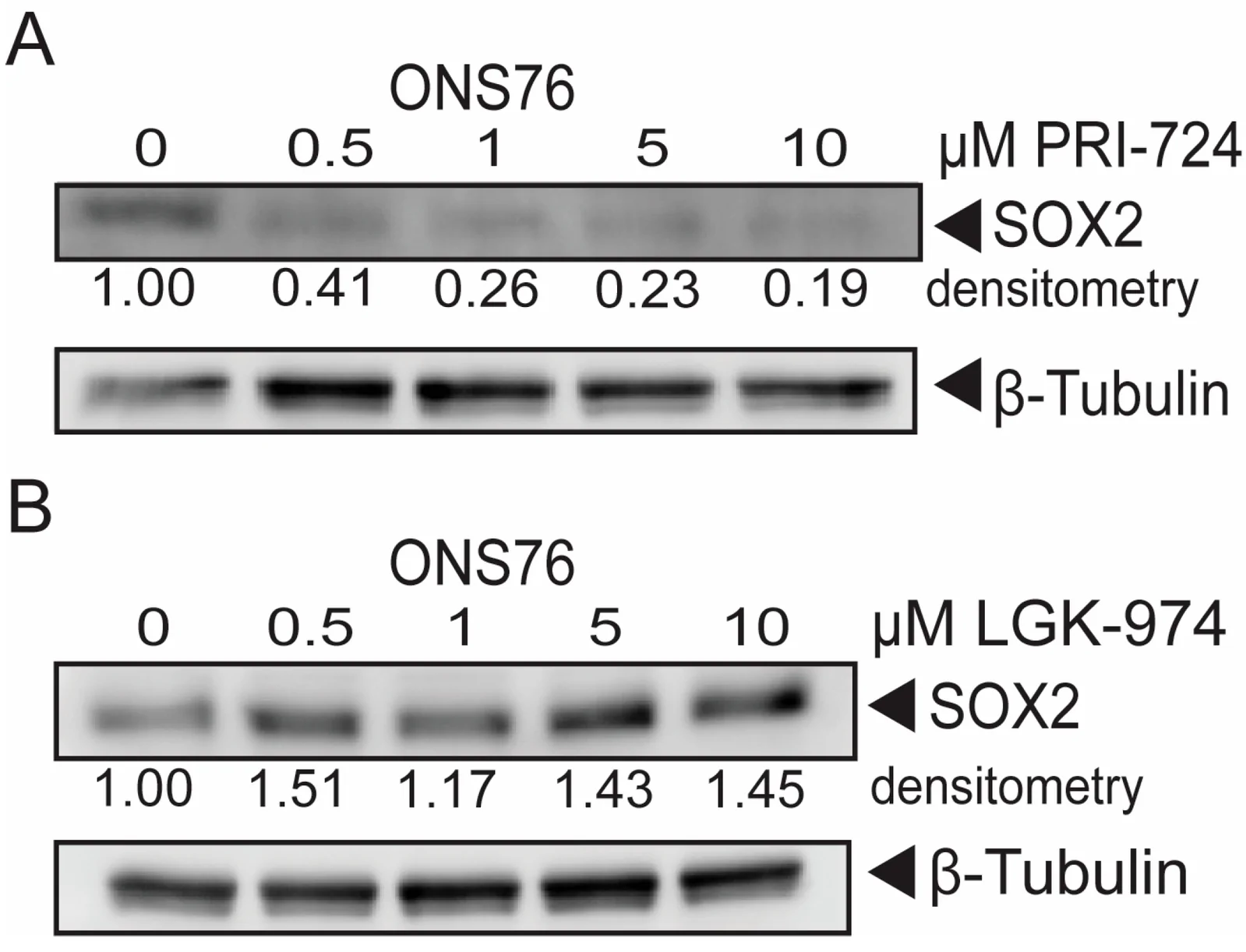
Effects of WNT inhibitors on SOX2 expression. Western blot analysis of whole-cell extracts to determine SOX2 protein levels in ONS76 sonic hedgehog medulloblastoma cells after 48 h of culture in the absence or presence of (**A**) PRI-724 or (**B**) LGK-974 at the indicated concentrations. β-Tubulin was used as a loading control. Uncropped blots for all westerns are provided in File S1. Similar results were obtained in a biological replicate using a different SOX2 antibody (CST #23064) for western blot analysis (File S1).

**Figure 2:**
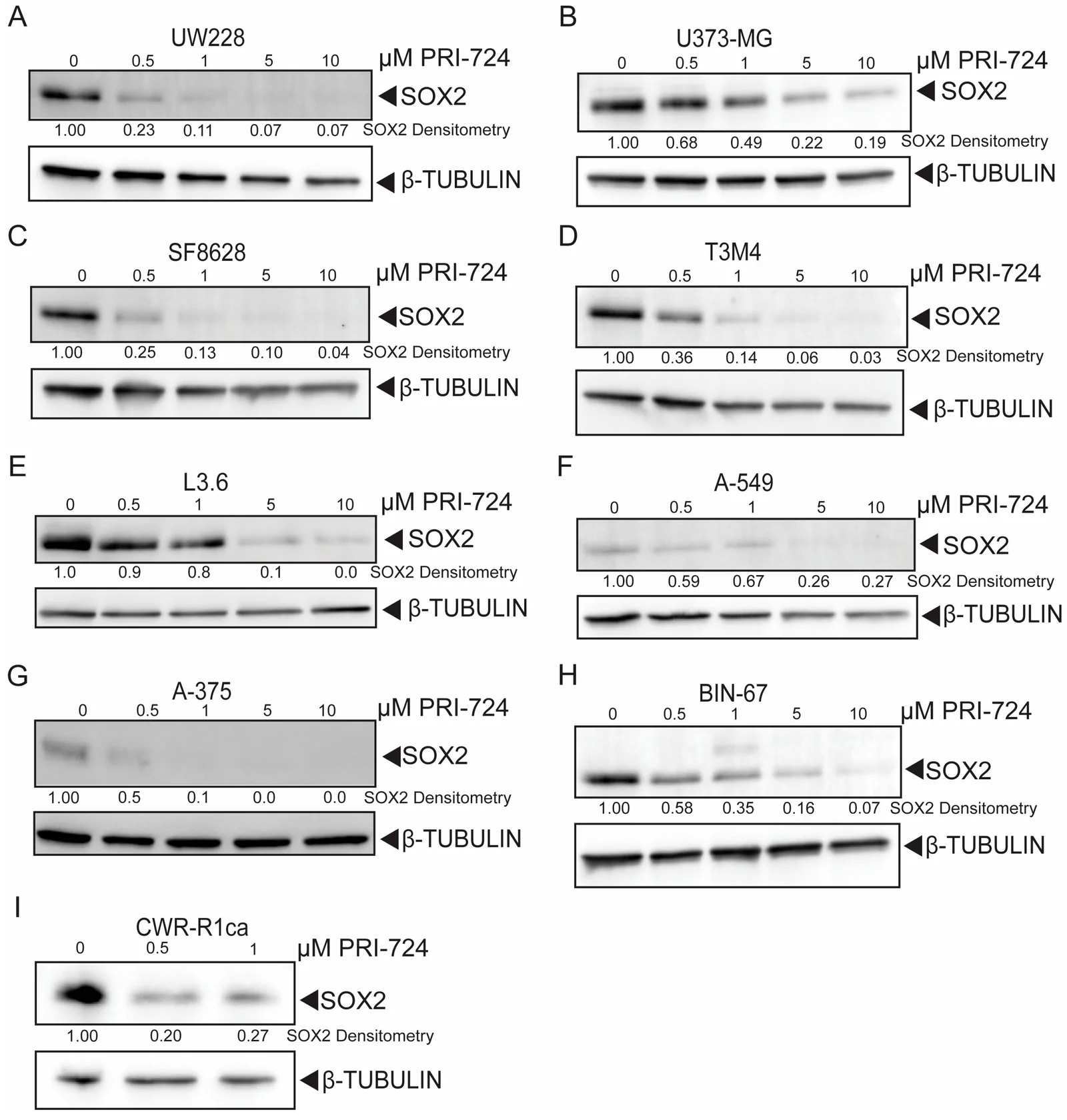
Effects of PRI-724 on SOX2 protein levels in 9 additional cell lines. Western blot analysis of relative SOX2 levels in whole cell extracts: (**A**) UW228 sonic hedgehog medulloblastoma, (**B**) U373-MG glioblastoma, (**C**) SF8628 diffuse intrinsic pontine glioma, (**D**) T3M4 and (**E**) L3.6 pancreatic ductal adenocarcinoma, (**F**) A-549 lung adenocarcinoma, (**G**) A-375 melanoma, (**H**) BIN-67 ovarian small cell carcinoma, and (**I**) CWR-R1ca prostate cancer cells after 48 h of culture in the absence and presence of PRI-724 at the indicated concentrations. (SOX2 decreases were not compared between cell lines). β-Tubulin was used as a loading control. Extracts from three of the cell lines (UW-228, A-375, and U373-MG glioblastoma) were tested with an additional SOX2 antibody (CST #23064), and similar results were obtained (File S1).

Treatment with PRI-724 resulted in a dose-dependent decrease in SOX2 expression, whereas treatment with LGK-974 failed to reduce SOX2 and appeared to slightly elevate SOX2 expression (Figure 1). Because PRI-724 induced a dose-dependent decrease in SOX2 protein expression in the ONS76 cells, we then evaluated PRI-724 in multiple SOX2-positive cancer cell lines to determine whether the effect of PRI-724 on SOX2 expression was consistent across a wide range of tumor cell lines. For these studies, we tested nine additional tumor cell lines that represent multiple tumor types. The tumor cell lines were UW228 (sonic hedgehog medulloblastoma), U373-MG (glioblastoma), SF8628 (diffuse intrinsic pontine glioma), T3M4 (pancreatic ductal adenocarcinoma), L3.6 (pancreatic ductal adenocarcinoma), A-549 (lung adenocarcinoma), A-375 (melanoma), BIN-67 (ovarian small cell carcinoma), and CWR-R1ca (prostate cancer). In each case, treatment of these cells with PRI-724 led to a dose-dependent decrease in SOX2 (Figure 2). While there were some differences in the sensitivity of the ten cell lines, there were substantial decreases in SOX2 in most of the cell lines even at 0.5 μM PRI-724. Taken together, these findings indicate that PRI-724 decreases SOX2 expression in a broad range of tumor cells, which represent eight different tumor types. In each tumor type, SOX2 is associated with poor clinical outcomes and/or cancer stem cell populations [4,18,19,49,50,51,52,53].

### 3.2. PRI-724 Blocks the Upregulation of SOX2 Expression in Tumor Cell Lines Induced by Therapeutic Cancer Regimens

**Figure 3:**
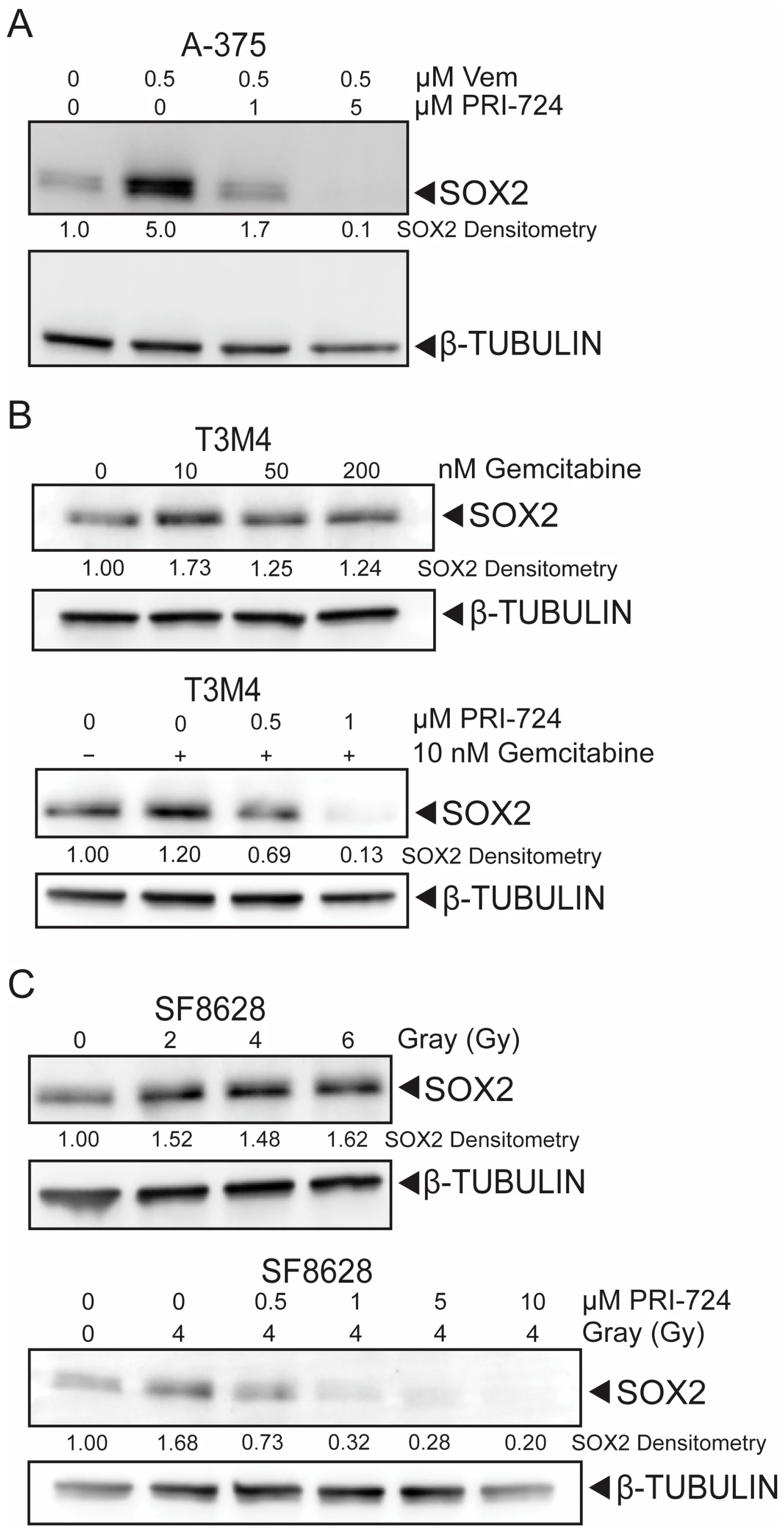
PRI-724 inhibition of therapy-induced upregulation of SOX2. (**A**) Western blot analysis of SOX2 protein levels in B-RAF-mutated A-375 cells after 48 h of culture in the absence or presence of vemurafenib (Vem; PLX4032) and PRI-724 at the indicated concentrations. (**B**) Western blot analysis of SOX2 protein levels in T3M4 cells after 72 h of culture in the absence or presence of Gemcitabine at increasing concentrations alone ((**B**), top), as well as Gemcitabine at 10 nM in combination with PRI-724 at the indicated concentrations ((**B**), bottom). (**C**) Western blot analysis of relative SOX2 protein levels in SF8628 cells 16 h after radiation exposure at increasing Gy ((**C**), top), followed by SOX2 western blot analysis of SF8628 isolates from cells cultured in the absence or presence of PRI-724 at the indicated concentrations for 48 h total with 4 Gy radiation exposure after the first 32 h of PRI-724 treatment and cell harvest 16 h later ((**C**), bottom). β-Tubulin was used as a loading control.

Several clinical cancer therapies have been shown to increase the expression of SOX2 [20,49]. To determine whether PRI-724 could block the upregulation of SOX2 caused by clinical treatments, we initially focused on the effect of vemurafenib (PLX4032). Previous studies have shown that vemurafenib, which is used to treat B-RAF mutant melanoma, upregulates SOX2 in the B-RAF mutant melanoma cell line A-375 [20]. As shown by others, treatment with vemurafenib increased SOX2 protein expression (Figure 3A). Significantly, treatment with both vemurafenib and PRI-724 not only blocked the upregulation of SOX2, but also reduced SOX2 expression below endogenous levels. Next, we tested the effects of gemcitabine, a DNA-damaging agent used in the treatment of pancreatic ductal adenocarcinoma. Previous studies have shown that gemcitabine increases the expression of SOX2 in pancreatic tumor cells and that SOX2 expression is increased in gemcitabine-resistant pancreatic tumor cells [18]. Similar to the results with vemurafenib, treatment of the pancreatic ductal adenocarcinoma cell line T3M4 with gemcitabine increased SOX2 expression, and this increase was blocked by co-treatment with PRI-724 to levels below those of endogenous SOX2 (Figure 3B). To extend these findings, we examined the effects of radiation, which has been shown to upregulate SOX2 expression in several tumor types [21,22,23,24,25,26]. For these studies, we tested the effects of radiation on SF8628 cells, which were derived from a diffuse intrinsic pontine glioma [54]. Patients with this cancer are inoperable due to the location of the tumor in the brainstem and are currently treated with radiation [55]. SF8628 cells were irradiated with 2 to 6 Grays of radiation. Higher doses of radiation were not tested to avoid extensive cell death. Sixteen hours after irradiation, cell extracts were prepared, and SOX2 protein levels were determined by western blot analysis. Sixteen hours was selected because previous studies had observed that SOX2 levels increased in glioblastoma cells between 12 and 24 h [56]. At each dose of radiation tested, 2–6 Grays, we observed an increase in the expression of SOX2 (Figure 3C). Importantly, treatment with PRI-724 blocked the upregulation of SOX2 by treatment with 4 Gray radiation. Moreover, there was a dose-dependent decrease in SOX2 in the irradiated cells, which was reduced below endogenous levels starting at 0.5 μM PRI-724. Thus, PRI-724 not only decreases the basal levels of SOX2, but it also prevents the upregulation of SOX2 induced by three commonly used clinical cancer treatments.

### 3.3. PRI-724 Decreases SOX2 at the RNA Level, but Not at the Protein Level, in Tumor Cell Lines

**Figure 4:**
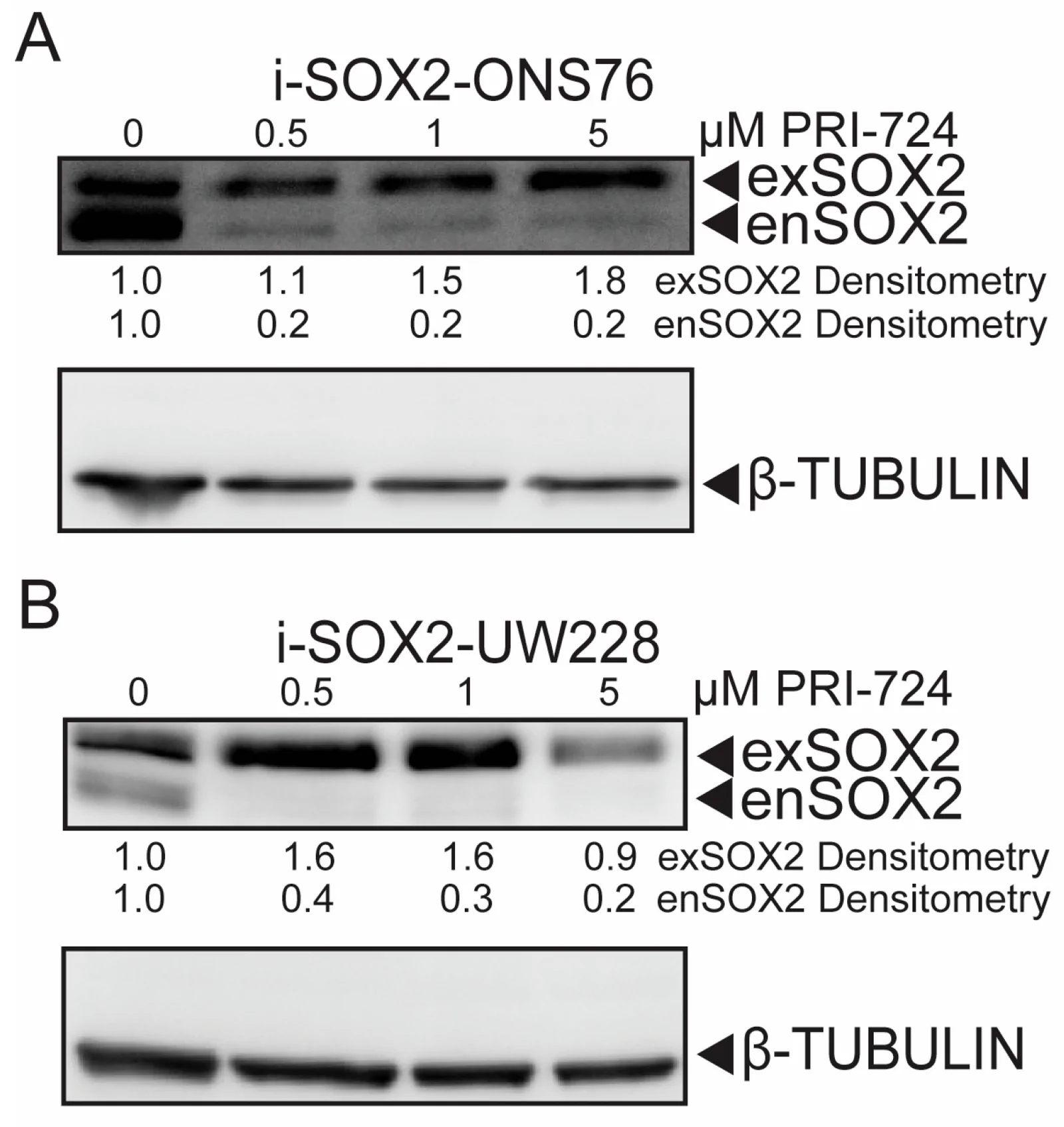
PRI-724 downregulation of endogenous SOX2 (enSOX2) but not exogenous SOX2 (exSOX2) in doxycycline-inducible cell lines. Western blot analysis of SOX2 protein levels in (**A**) i-SOX2-ONS76 or (**B**) i-SOX2-UW228 cells after 48 h of culture in the absence or presence of PRI-724 at the indicated concentrations. β-Tubulin was used as a loading control.

**Figure 5:**
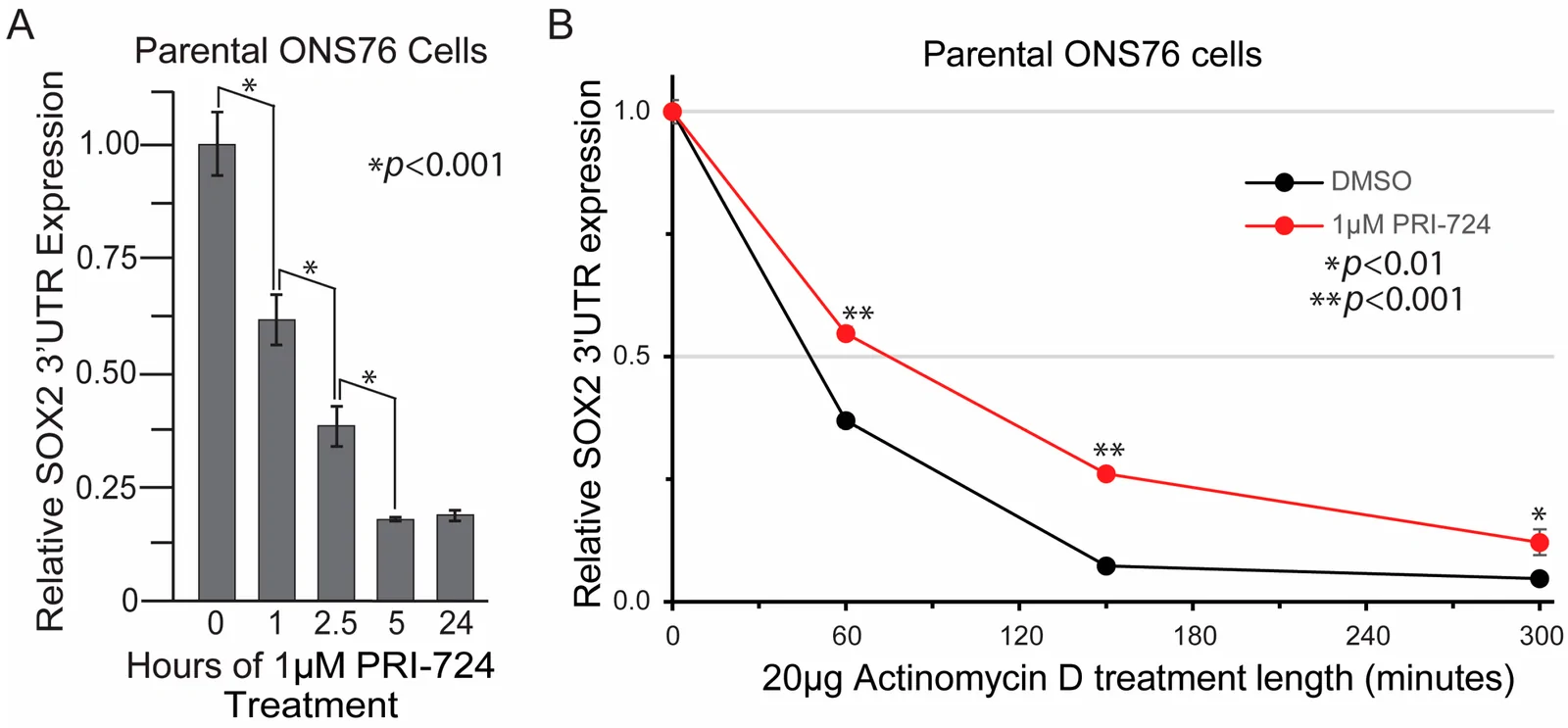
Effects of PRI-724 on *SOX2* RNA expression. (**A**) RT-qPCR analysis of *SOX2 3’UTR* expression relative to *GAPDH* in ONS76 cells after time-course treatment in the absence and presence of PRI-724 at the indicated concentrations. (**B**) RT-qPCR analysis of *SOX2 3’UTR* expression relative to *GAPDH* in ONS76 cells in the absence or presence of 1 µM PRI-724 for 16 h prior to treatment with 20 µg of Actinomycin D for the indicated time points. Error bars represent standard deviation, and *p*-values were determined by a two-tailed *t*-test for two samples with equal variance, *n* = 3.

Next, we focused on the molecular mechanisms responsible for the downregulation of SOX2 by PRI-724. To evaluate how PRI-724 decreases SOX2 protein expression, we initially examined whether PRI-724 reduced the protein stability of SOX2. For these experiments, we used two SOX2 doxycycline-inducible cell lines (i-SOX2-ONS76 and i-SOX2-UW228), which express both endogenous SOX2 and low levels of exogenous Flag-tagged SOX2 due to leaky expression of SOX2 from the transgene in the absence of doxycycline. The i-SOX2-ONS76 and i-SOX2-UW228 cells enabled us to monitor the effects of PRI-724 on both endogenously and exogenously expressed SOX2. Treatment of both cell lines with PRI-724 led to a reduction in endogenously expressed SOX2, with little or no effects on exogenously expressed SOX2 (Figure 4). Importantly, these findings indicate that PRI-724 has little, if any, effect on SOX2 protein stability. Next, we examined whether the decrease in endogenous SOX2 protein was due to changes in *SOX2* mRNA levels. For these studies, *SOX2* mRNA was monitored by RT-qPCR. We determined that treatment with 1 µM of PRI-724 reduced *SOX2* mRNA expression by ~80% within five hours (Figure 5A). As reported below in our RNA-seq analysis, SOX2 expression is also reduced at the 5 h time point in UW228 and SF8628 cells. To extend these findings, we examined the effects of PRI-724 on the stability of *SOX2* RNA using ONS76 cells. For this purpose, the cells were treated with Actinomycin D, which inhibits transcription, in the presence and absence of PRI-724 (Figure 5B). Surprisingly, *SOX2* RNA decreased more slowly in the cells pre-treated with PRI-724 followed by actinomycin D than in cells treated with actinomycin D on its own, which suggests that PRI-724 may slightly increase the stability of *SOX2* RNA. Taken together, these findings indicate that PRI-724 reduces *SOX2* expression at the RNA level, without significantly reducing SOX2 protein or mRNA stability.

### 3.4. PRI-724 Induces a Reversible State of Cellular Growth Arrest in ONS76 Medulloblastoma Cells

**Figure 6:**
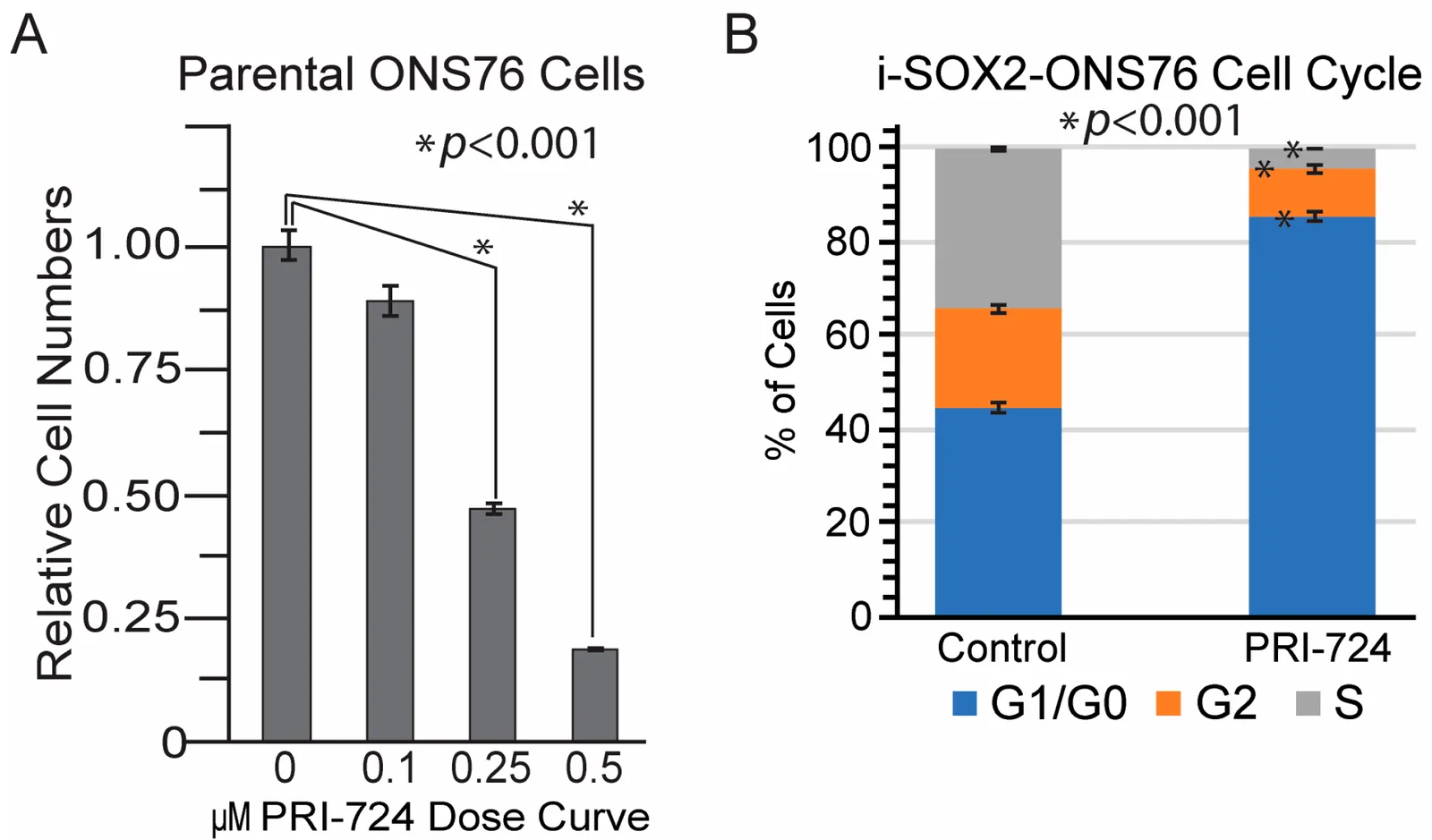
Effects of PRI-724 on proliferation and the cell cycle. (**A**) Proliferation of ONS76 cells as determined by direct cell counting with a Z1 Coulter^®^ Particle Counter following 72 h of culture in the absence or presence of PRI-724 at the indicated concentrations was performed in triplicate. In a separate experiment, these results were verified by performing an MTT assay. (**B**) Cell cycle analysis of i-SOX2-ONS76 cells was performed by flow cytometry after 4 days of culture in the absence or presence of 1 µM PRI-724. Error bars represent standard deviation, and *p*-values were determined by a two-tailed *t*-test for two samples with equal variance, *n* = 3.

To better understand the effects of PRI-724 on the growth of ONS76 cells, we initially examined its effects on cell proliferation during short-term treatment. Treatment with PRI-724 over a three-day period led to a dose-dependent decrease in cell number (Figure 6A), which was verified by performing an MTT assay. To further analyze the growth inhibitory properties, cell cycle analysis was performed (Figure 6B). Treatment with PRI-724 led to a roughly two-fold increase in the proportion of cells within the G1/G0 phase of the cell cycle. Additionally, there was close to a ninety percent decrease in the proportion of cells within the S phase and an approximately fifty percent decrease in the number of cells within the G2 phase of the cell cycle.

**Figure 7:**
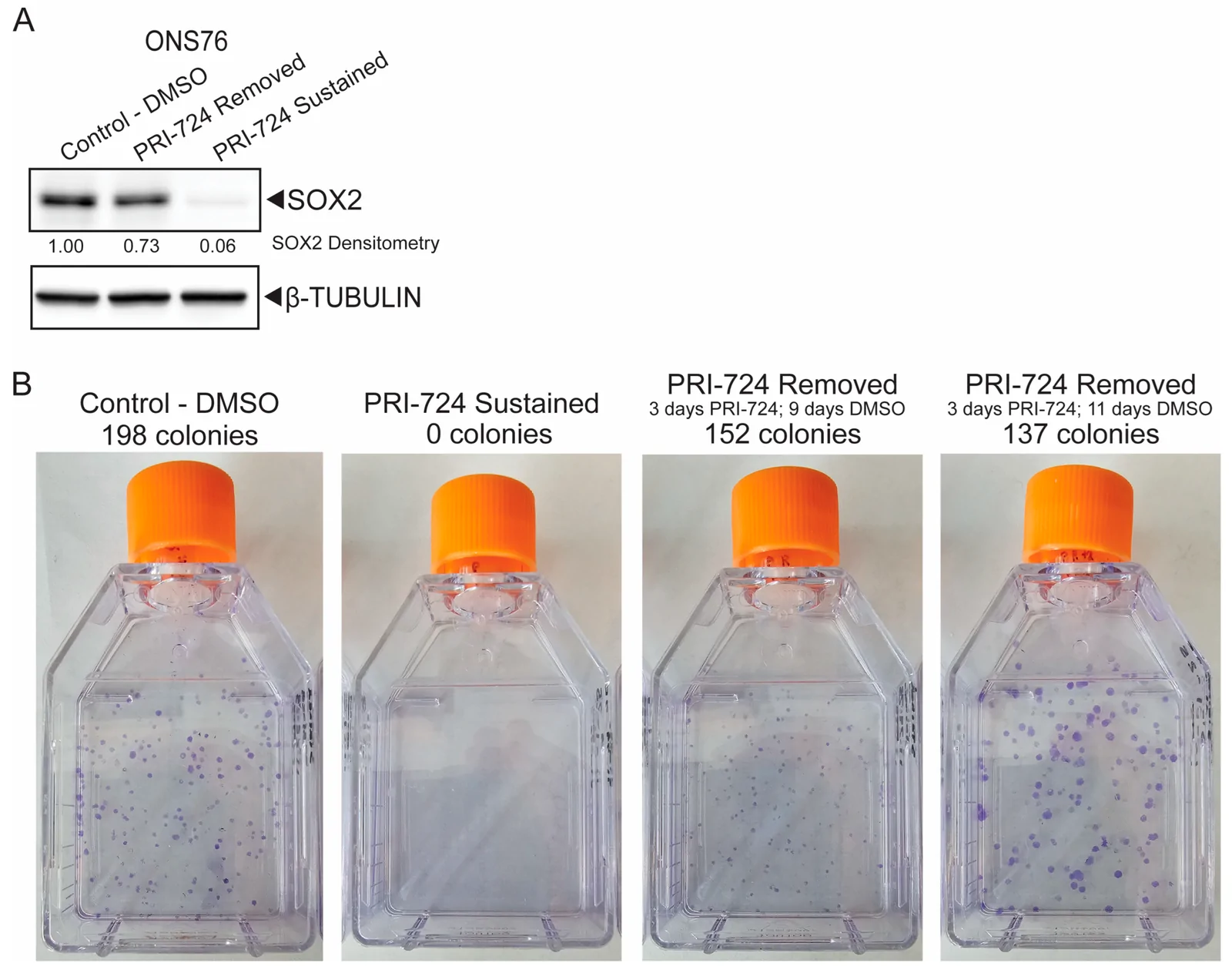
Reversible effects of PRI-724 on SOX2 protein levels and proliferation. (**A**) Western blot analysis of SOX2 protein levels in ONS76 cells cultured in the absence (Control–DMSO) or presence of 1 µM PRI-724 for 48 h before an additional 72 h of culture in the absence (Control–DMSO and PRI-724 Removed) or presence of 1 µM PRI-724 (PRI-724 Sustained). (**B**) Colony formation assay of ONS76 cells under four different conditions. The control flask was maintained in DMSO-containing medium for 9 days. The PRI-724 sustained flask was continually maintained in 1 µM PRI-724-containing medium. Then, two flasks to test the reversibility of PRI-724 effects on proliferation were cultured in the presence of 1 µM PRI-724 for 72 h before the removal of PRI-724 and then culturing in DMSO-containing medium for 9 days or 11 days before processing and counting colonies.

Next, we examined whether the effects of PRI-724 on SOX2 protein expression and cell proliferation (clonal growth) were reversible by removing PRI-724. Removal of PRI-724 after a two-day treatment of ONS76 cells with the drug led to substantial recovery of SOX2 expression (Figure 7A). Equally importantly, removal of PRI-724 led to renewed cell proliferation, as judged by the clonal growth of ONS76 cells (Figure 7B). In contrast, sustained treatment with PRI-724 over the same timeframe did not result in recovery of SOX2 protein expression and completely blocked clonal growth. Overall, these results indicate that PRI-724 acts primarily as a cytostatic agent by inducing reversible G1/G0 growth arrest of ONS76 cells.

### 3.5. Effects of PRI-724 on the Transcriptome in Three Pediatric Brain Tumor Cell Lines

**Figure 8:**
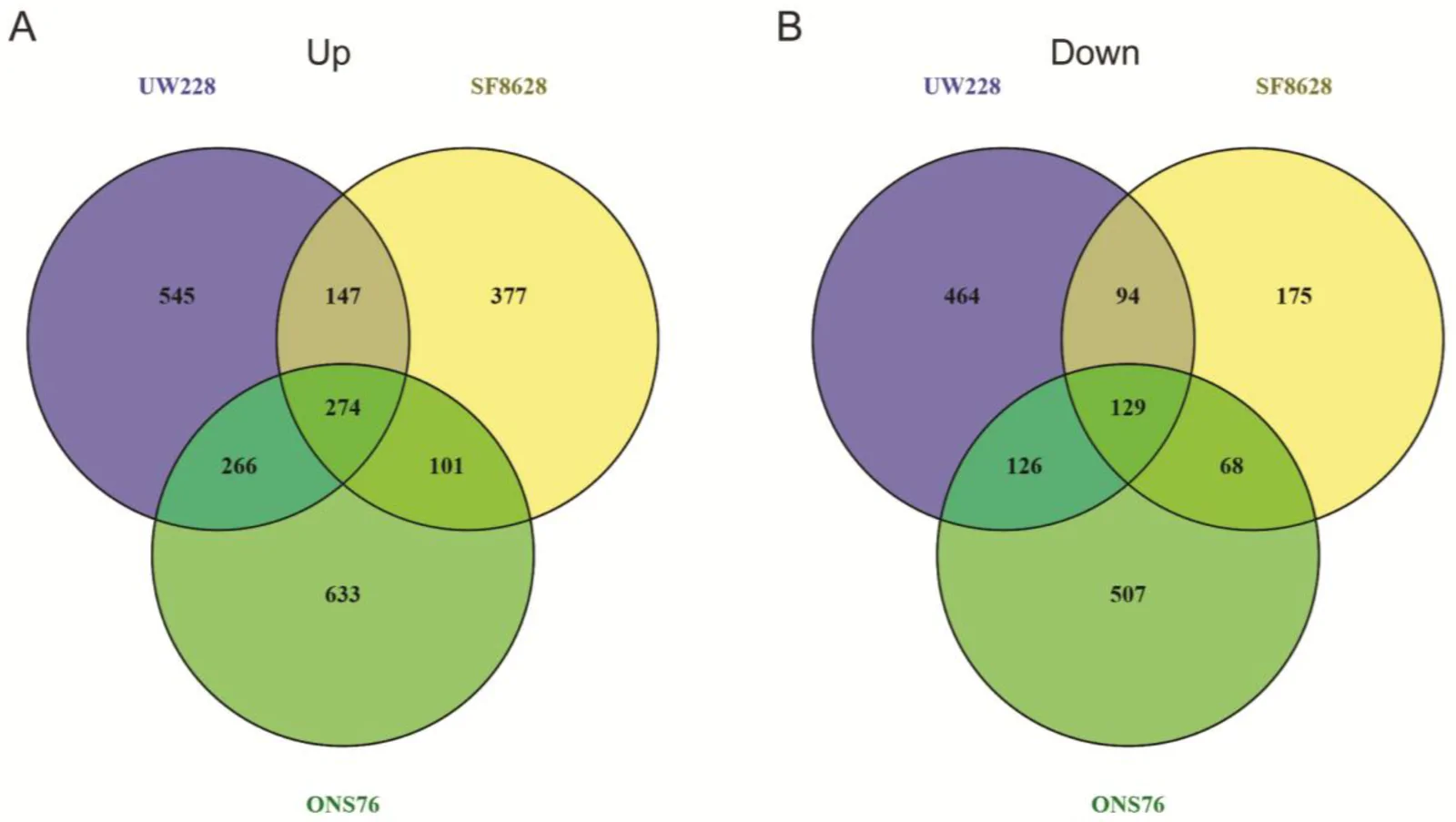
Venn diagram of differentially expressed genes in common between the ONS76, UW228, and/or SF8628 cells. (**A**) Common upregulated genes in cells treated with PRI-724. (**B**) Common downregulated genes in cells treated with PRI-724. The Venn diagram was created using Venny 2.1 [45]. Genes were filtered from mRNA-sequencing data conducted by Novogene and filtered for padj < 0.05 and Log2FC > 0.5 or <−0.5.

PRI-724 clearly downregulates SOX2 in a broad spectrum of tumor cell types. Given its ability to disrupt the interaction between β-Catenin and CBP [40,57,58], we suspected that PRI-724 would also alter the expression of a large number of genes. To determine the extent to which PRI-724 alters the transcriptome, and to determine whether it alters the expression of a common set of genes in several cell types, we performed RNA-seq. For this purpose, we selected three pediatric brain tumor cell lines rather than a more diverse set of tumor types. Specifically, we selected ONS76, UW228, and SF8628 cells for this analysis. ONS76 cells and UW228 cells were selected because they are Sonic Hedgehog medulloblastoma cell lines that are wild-type and TP53 mutant, respectively. SF8628, which is a diffuse intrinsic pontine glioma cell line, was included in this study because it is another pediatric brain tumor, which is very difficult to treat. Each of the three brain tumor cell lines was treated with and without PRI-724 for 5 h (Figure 8). The 5 h time point was selected because we wanted to focus on the initial effects of PRI-724 and because PRI-724 maximally reduced *SOX2* mRNA expression at 5 h (Figure 5A). As expected, RNA-seq analysis demonstrated that treatment of the cells with PRI-724 led to substantial downregulation of multiple genes, including decreased *SOX2* expression, in each of the three cell lines. Over 800 genes in both ONS76 and UW228 cells and over 450 genes in SF8628 cells were downregulated by PRI-724 after 5 h. Unexpectedly, treatment with PRI-724 led to an even larger upregulation of genes. Over 1200 genes in ONS76 and UW228 cells and nearly 900 genes were upregulated in SF8628 cells. Hence, treatment with PRI-724 significantly alters the transcriptome of each of the three pediatric brain tumor cell lines.

**Figure 9:**
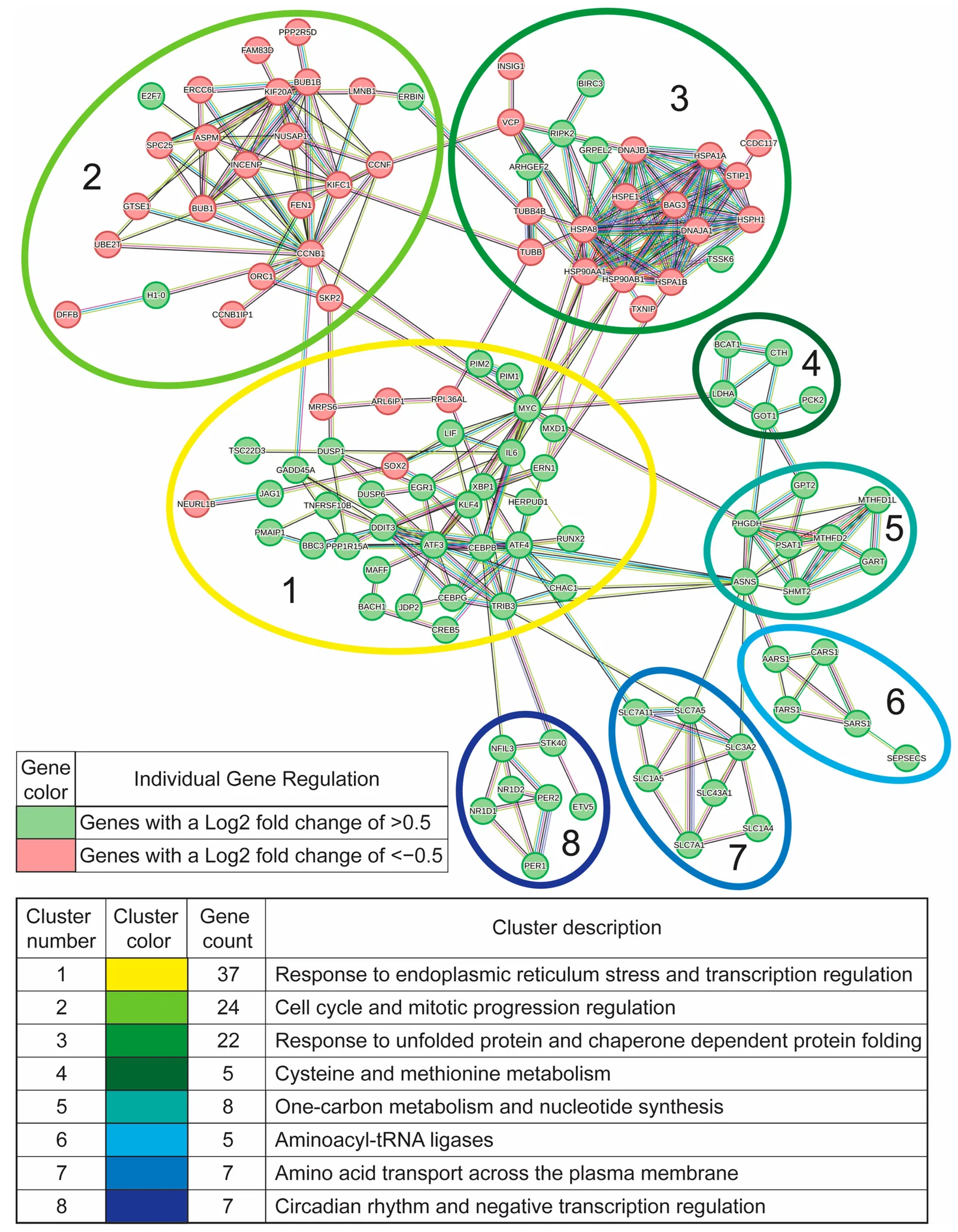
STRING [46] map of the commonly altered genes after treatment of ONS76, UW228, and SF8628 pediatric brain tumor cell lines with PRI-724. Small, solid circles represent proteins of commonly upregulated (green) and downregulated (red) genes, and the lines represent connections. Clusters of genes are color-coded and circled, with descriptions for each cluster provided in the figure key. The mapping parameters were set to a minimum interaction score of 0.7 (High Confidence), and the unconnected nodes and clusters were removed. Clusters were decided by an MCL clustering inflation parameter of 1.4. Cluster descriptions were determined from the top Gene Ontology Biological Processes for each individual cluster.

A shared set of 274 upregulated genes and 129 downregulated genes were identified among all three cell lines following PRI-724 treatment (Supplementary Table S1). As expected, *SOX2* was among the downregulated genes common to all three cell lines. To understand the function of the common set of upregulated and downregulated genes, we performed network analysis using the STRING database (Figure 9). Of the 403 commonly altered genes, 288 genes were not shown in the STRING network map because they did not fit the high-confidence interaction criteria as described in the Materials and Methods section, or they exhibited low-significance interactions with known networks. Analysis of the remaining 115 commonly altered genes identified eight gene clusters, which were assigned a general description by the network tool based on the GO enrichment analysis. Of these eight clusters (Figure 9), six primarily consisted of upregulated genes (clusters 1 and 4–8) and two primarily consisted of downregulated genes (clusters 2 and 3). Notably, three of the upregulated gene clusters consist of genes associated with amino acid transport (Figure 9, cluster 7), tRNA ligases (cluster 6), and metabolism of amino acids (cluster 4). Interestingly, the upregulation of *SLC7A11* and *SLC3A2* (cluster 7), which promotes the uptake of cystine [59], and the upregulation of genes in cluster 4 (categorized in cysteine and methionine metabolism) are consistent with a lower ferroptosis profile of the PRI-724-treated cells. Conversely, the largest cluster (cluster 1), which includes decreased *SOX2* and increases in *ATF3*, *ATF4*, *CHAC1* and *DDIT3*, likely indicates increased endoplasmic reticulum (ER) stress [60]. This is even more interesting because decreases in cluster 3, which involves *DNAJB*, *HSP90AA1*, and *HSPA8*, may reflect an unfolded protein response and decreased chaperone-dependent protein folding [61]. Together, these results indicate PRI-724 has a wide-reaching impact on the transcriptome, including changes in expression of genes related to stress response pathways.

## 4. Discussion

SOX2 is recognized as a bona fide therapeutic target because of its critical roles in cancer stem cell populations and its expression in over 20 different types of human cancer [3]. Equally important, SOX2 is implicated directly in drug resistance in multiple tumor types [15,16,17,18,19,20]. In this study, we demonstrate that the downstream WNT inhibitor PRI-724 (Foscenvivint), but not the upstream WNT inhibitor LGK-974, strongly decreases SOX2 expression. Currently, it is unclear why LGK-974 did not inhibit SOX2 expression. However, LGK-974 is known to block release of WNT ligands [47], whereas PRI-724 is reported to block the interaction between CBP and β-catenin [41]. Consequently, SOX2 expression in the cells tested may not be dependent on their release of WNT ligands, but is likely to be dependent on the downstream interaction between CBP and β-catenin. Importantly, we show that PRI-724 downregulates SOX2 in 10 different tumor cell lines that represent eight different tumor types: three different types of brain cancer, pancreatic ductal adenocarcinoma, lung cancer, melanoma, prostate cancer, and clear cell ovarian cancer. Given the effects of PRI-724 on SOX2 expression in such a broad spectrum of tumor cell types, and reports that PRI-724 blocks the interaction between β-Catenin and the co-activator CBP [40,57,58], we suggest there is a critical WNT/SOX2 signaling axis that is active in many, if not most, SOX2-positive cancers. Equally interesting, there are reports that SOX2 influences WNT signaling [62], and we have found that elevating SOX2 increases the expression of genes that downregulate WNT signaling [14]. Thus, an important regulatory loop appears to exist between SOX2 and WNT signaling.

In the case of ONS76 cells, we show that PRI-724 appears to have little, if any, effect on SOX2 protein stability. Rather, PRI-724 rapidly downregulates *SOX2* RNA. Significantly, PRI-724 did not downregulate *SOX2* by decreasing its RNA stability. Consequently, it appears that PRI-724 downregulates *SOX2* at the transcriptional level, which is consistent with its reported mechanism of action noted above. We also determined that PRI-724 leads to a reversible state of G0/G1 growth arrest. Specifically, removal of PRI-724 after three days leads to the recovery of both SOX2 protein expression and clonal growth. Thus, PRI-724 appears to be cytostatic rather than cytotoxic. Consequently, PRI-724 may slow the growth of SOX2-positive cancers, but, used on its own, it is unlikely to effectively eradicate SOX2-positive tumor cells.

Rather than working as a sole therapeutic agent, our findings suggest PRI-724 could be effective when combined with other therapies. In our study, we determined that the upregulation of SOX2 by vemurafenib, gemcitabine, and irradiation is each blocked by PRI-724. Specifically, we show that PRI-724 not only reduces endogenous SOX2 expression but also blocks the upregulation of SOX2 by these three therapeutic treatments. In the case of vemurafenib, which is used to treat B-Raf mutant melanoma, it is possible that simultaneous treatment with both vemurafenib and PRI-724 could either prevent or delay the invariant drug resistance observed when melanoma patients are treated with vemurafenib on its own [63]. In this connection, a recent study reported that PRI-724 induced apoptosis in vemurafenib- and trametinib-resistant melanoma cells [64]. Thus, we believe further study of PRI-724 in combination with vemurafenib is warranted. We speculate that treatment-induced upregulation of SOX2, a known pioneering transcription factor, is likely to promote increased chromatin accessibility and, as a result, increased tumor cell plasticity and drug resistance. Similarly, combining PRI-724 and radiotherapy could enhance the radiosensitivity of difficult-to-treat cancers, such as diffuse intrinsic pontine gliomas [55]. Patients with this cancer have a five-year survival that approaches 1% [55]. Due to the location of these tumors in the brainstem, these tumors are inoperable and are currently treated with radiation. Interestingly, PRI-724 has been tested in clinical trials, including one where it was used in combination with gemcitabine for metastatic pancreatic adenocarcinoma [41]. In this trial, PRI-724 in combination with gemcitabine was found to have a low toxicity profile, but only modest improvement in efficacy [41]. It is possible that the combination of gemcitabine and PRI-724 may work best for the subset of pancreatic cancer patients whose tumors express elevated levels of SOX2, given that pancreatic cancer patients with elevated levels of SOX2 have a shorter progression-free survival [18]. More generally, PRI-724 could be effective when combined with a range of therapeutic regimens that upregulate SOX2 expression and ultimately culminate in reduced responses to radiation and drugs used clinically.

Finally, our studies show that PRI-724 substantially alters transcription well beyond its effects on SOX2. Our RNA-Seq data generated from three pediatric brain tumor cell lines determined that treatment with PRI-724 substantially alters the transcriptome, including downregulation of *SOX2* in all three cell lines. In the cases of ONS76 and UW228 medulloblastoma cells, PRI-724 altered the expression of over 2000 genes at the RNA level, including the expression of genes associated with stress response pathways, in particular ER stress response. Importantly, our results suggest that combining PRI-724 with therapeutics that enhance ER stress could be productive. In this regard, treatment with PRI-724 decreased the expression of genes associated with unfolded protein and chaperone-dependent protein folding, while increasing genes associated with ER stress. Thus, we suggest that the use of PRI-724 in combination with other clinically used therapies could provide significant clinical benefit. In this connection, a recent study reported that the precursor to PRI-724, ICG-001, significantly enhanced the efficacy of CAR-T cell therapy in a glioma mouse model [65].

## 5. Conclusions

In conclusion, the findings described in this study present a novel strategy to target SOX2 expression across eight different cancer types. Additionally, we show that PRI-724 modulates the expression of genes associated with stress response pathways, while effectively decreasing SOX2 expression in the presence of clinical treatments that upregulate expression of SOX2 on their own. Going forward, it will be important to test whether PRI-724 reduces the expression of SOX2 in vivo by using xenografts formed after engraftment of cell lines used in this study and/or in genetically modified mouse models known to express SOX2. Moreover, it will be important to determine whether PRI-724 used in combination with vemurafenib, gemcitabine, or radiation alters the survival of melanoma, pancreatic ductal adenocarcinoma cells, or diffuse intrinsic pontine glioma cells, respectively. In this regard, it will also be important to test whether PRI-724 can prevent or delay the onset of gemcitabine-resistant pancreatic ductal adenocarcinoma cells and vemurafenib-resistant melanoma cells. Similarly, it should be determined whether PRI-724 can prevent or delay the generation of radiation-resistant diffuse intrinsic pontine glioma cells. Lastly, this study did not examine whether the growth inhibitory effects of PRI-724 are solely due to the knockdown of SOX2. Given the large effects of PRI-724 on the transcriptome, we suspect that this is not the case.

## Figures and Tables

**Table 1:** Primers used for RT-qPCR.

Name	Forward Primer Sequence 5′-3′	Reverse Primer Sequence 5′-3′
*GAPDH*	ACAGCGACACCCACTCCTCC	GAGGTCCACCACCCTGTTGC
*SOX2 3* *′* *UTR*	GGAGAAAACCCGGTACGCTC	AGTTTTCTTGTCGGCATCGC

## Data Availability

Raw and processed RNA-seq data files have been deposited at the Gene Expression Omnibus under the accession number GSE347454.

## Supplementary Materials

The following supporting information can be downloaded at: https://www.mdpi.com/article/10.3390/cancers18213569/s1, Table S1: Common Genes List; File S1: Original western blots.

## Abbreviations

The following abbreviations are used in this manuscript:

ATF3	Activating transcription factor 3
ATF4	Activating transcription factor 4
B-RAF	B-Raf murine sarcoma viral oncogene homolog B
CAR-T	Chimeric antigen receptor T
CBP	CREB-binding protein; CREB is cAMP response element-binding protein
CD44	Cluster of differentiation 44
cDNA	Complementary deoxyribonucleic acid
CHAC1	ChaC Glutathione Specific Gamma-Glutamylcyclotransferase 1
DDIT3	DNA Damage Inducible Transcript 3
DMSO	Dimethyl sulfoxide
DNAJB	DnaJ Heat Shock Protein Family (Hsp40) Member B1
ER	Endoplasmic Reticulum
FPKM	Fragments per kilobase per million mapped reads
GAPDH	Glyceraldehyde-3-Phosphate Dehydrogenase
GO	Gene ontology
Gray	Gy
H3K27M	Histone H3 genetic mutation where lysine 27 is replaced by methionine
HISAT2	Hierarchical Indexing for Spliced Alignment of Transcripts 2
HSP90AA1	Heat Shock Protein 90 Alpha Family Class A Member 1
HSPA8	Heat Shock Protein Family A (Hsp70) Member 8
mRNA	Messenger ribonucleic acid
MTT	3-(4,5-dimethylthiazol-2-yl)-2,5-diphenyltetrazolium bromide
PORCN	Protein-serine O-palmitoleoyltransferase porcupine
RIPA	Radioimmunoprecipitation assay buffer
RPMI	Roswell Park Memorial Institute
RT-qPCR	Real-time quantitative polymerase chain reaction
SLC7A11	Solute Carrier Family 7 Member 11
SLC3A2	Solute Carrier Family 3 Member 2
SOX	SRY-related HMG-box
STRING	Search Tool for the Retrieval of Interacting Genes/Proteins
WNT	Wingless/Integrated
