# Supplementary material for: The WNT Inhibitor PRI-724 Depletes Endogenous SOX2 and Blocks SOX2 Upregulation by Therapeutic Regimes
Source: Cancers (Basel). 2026 Nov 6;18(21):3569. doi: 10.3390/cancers18213569 (PMC13762130; doi:10.3390/cancers18213569)
Supplement: Supplementary file 1 [file cancers-18-03569-s001.zip › cancers-4611361-File S1-Original-western-blots.pdf]

Figure 1

A

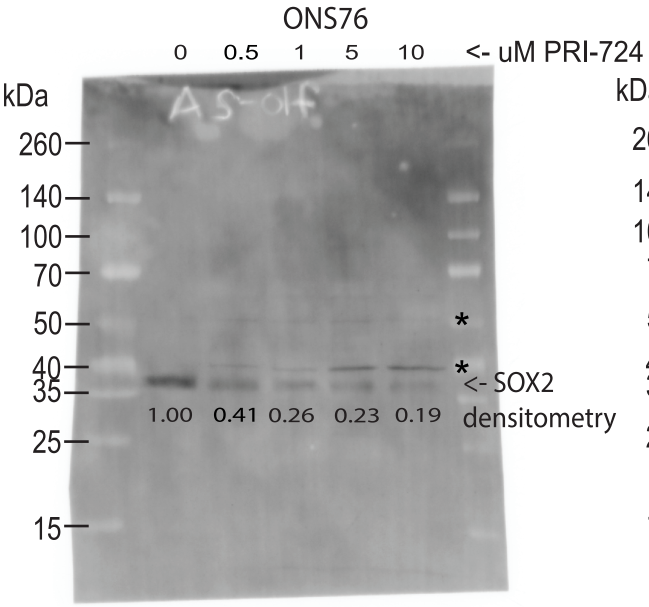

1:1000 SOX2 (CST 3579S)
1:5000 anti-rabbit secondary (CST 7054S)

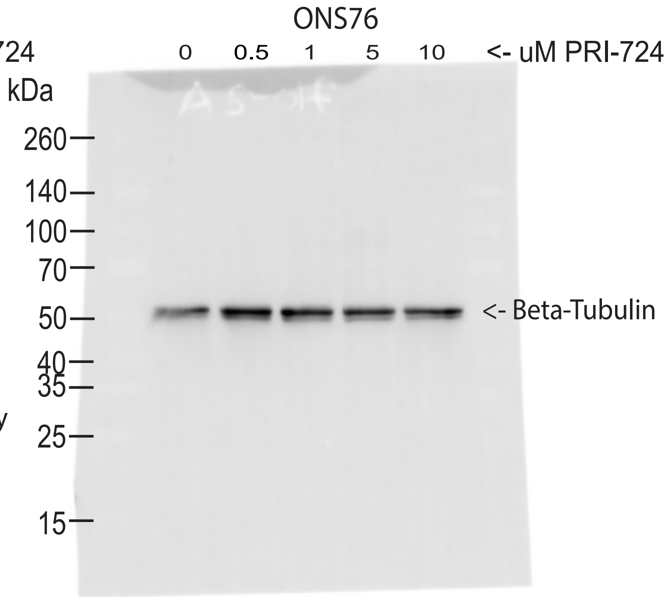

1:1000 β-TUBULIN (CST 2146S)
1:5000 anti-rabbit secondary (CST 7054S)

*Appears to be a non-specific band. We were unable to rule out the possibility of a post-translational modification.

B

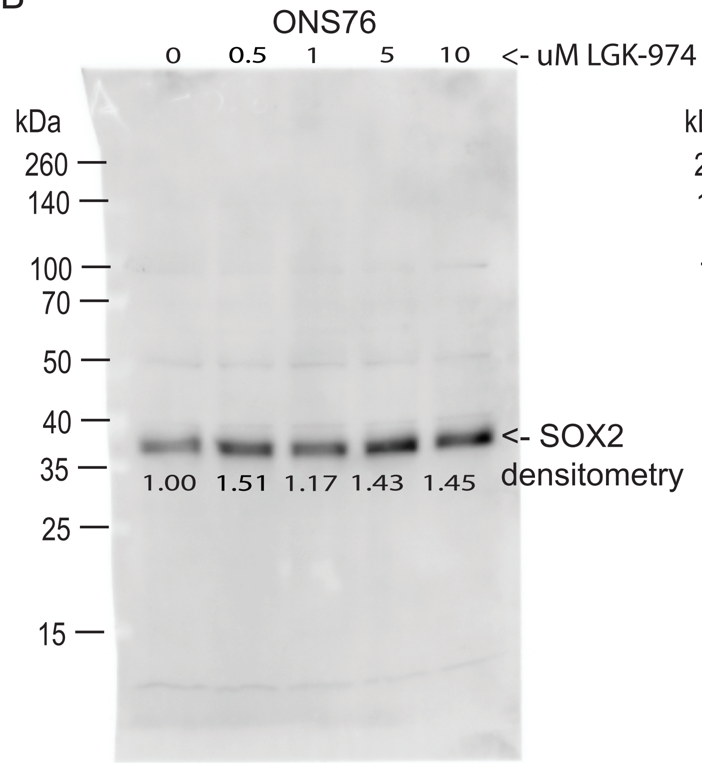

1:1000 SOX2 (CST 3579S)
1:5000 anti-rabbit secondary (CST 7054S)

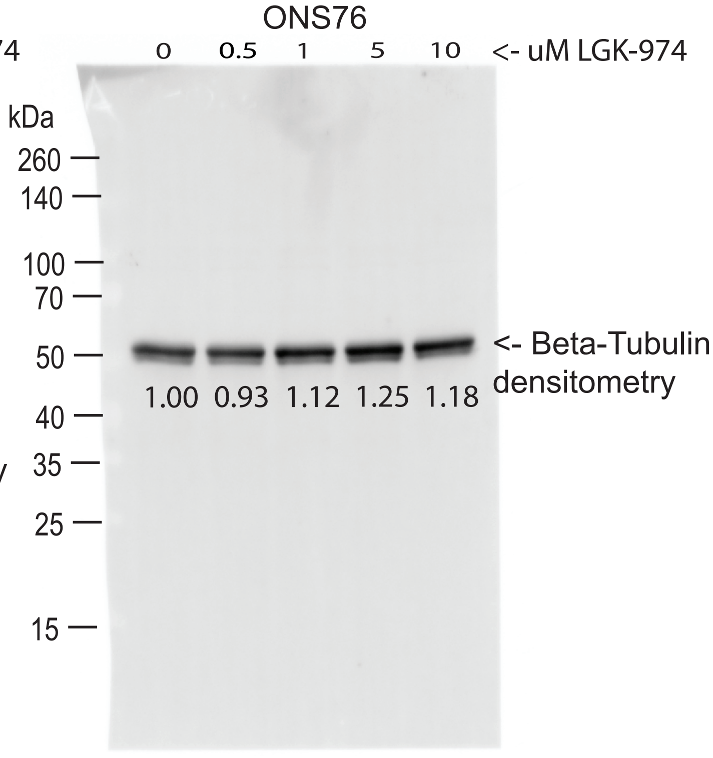

1:1000 β-TUBULIN (CST 2146S)
1:5000 anti-rabbit secondary (CST 7054S)

Figure 1 - Biological Replicate; incubated with a different SOX2 antibody

A

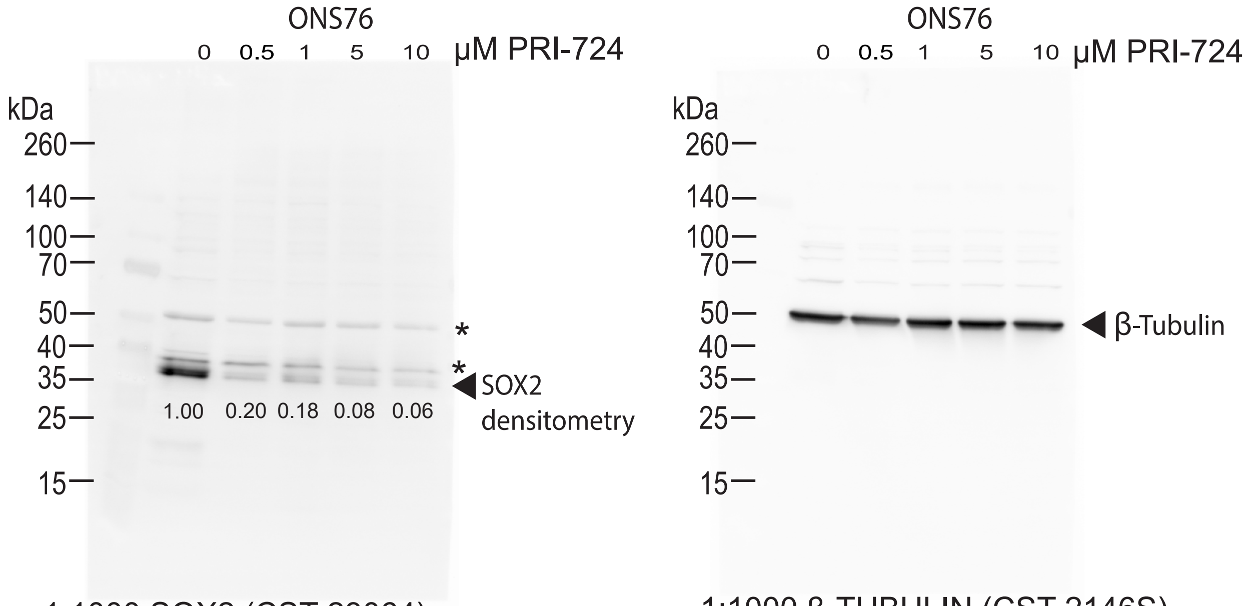

1:1000 SOX2 (CST 23064)
1:5000 anti-rabbit secondary (CST 7054S)

1:1000 β-TUBULIN (CST 2146S)
1:5000 anti-rabbit secondary (CST 7054S)

*Appears to be a non-specific band. We were unable to rule out the possibility of a post-translational modification.

B

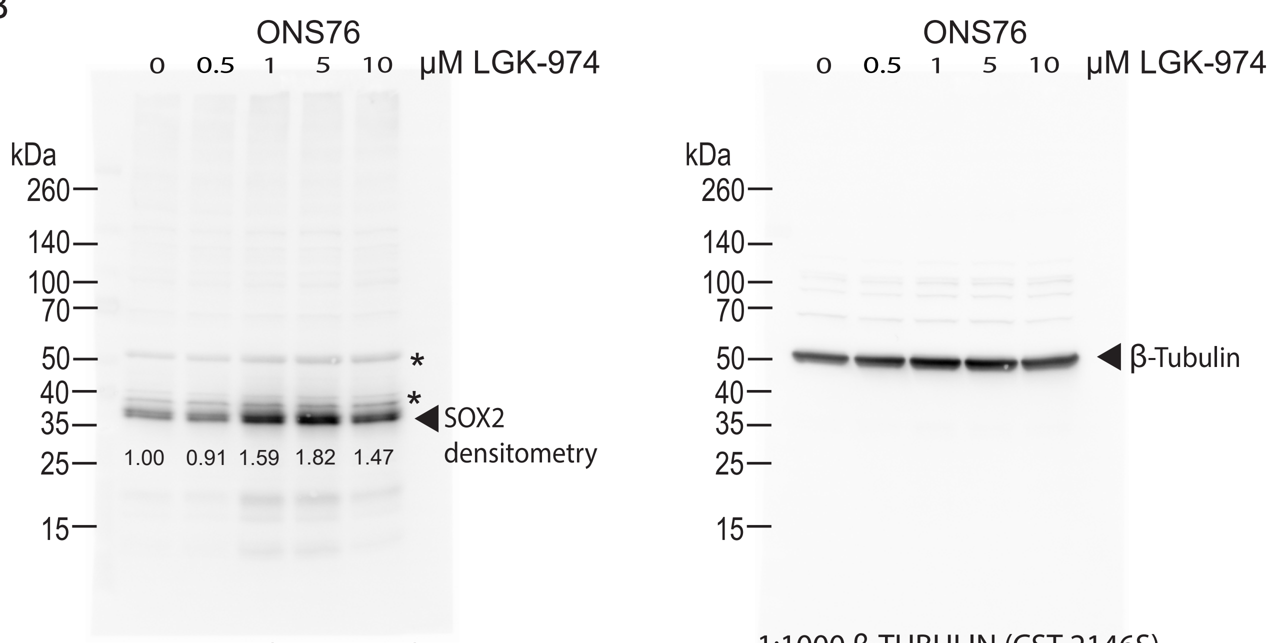

1:1000 SOX2 (CST 23064)
1:5000 anti-rabbit secondary (CST 7054S)

1:1000 β-TUBULIN (CST 2146S)
1:5000 anti-rabbit secondary (CST 7054S)

*Appears to be a non-specific band. We were unable to rule out the possibility of a post-translational modification.

Figure 2

A

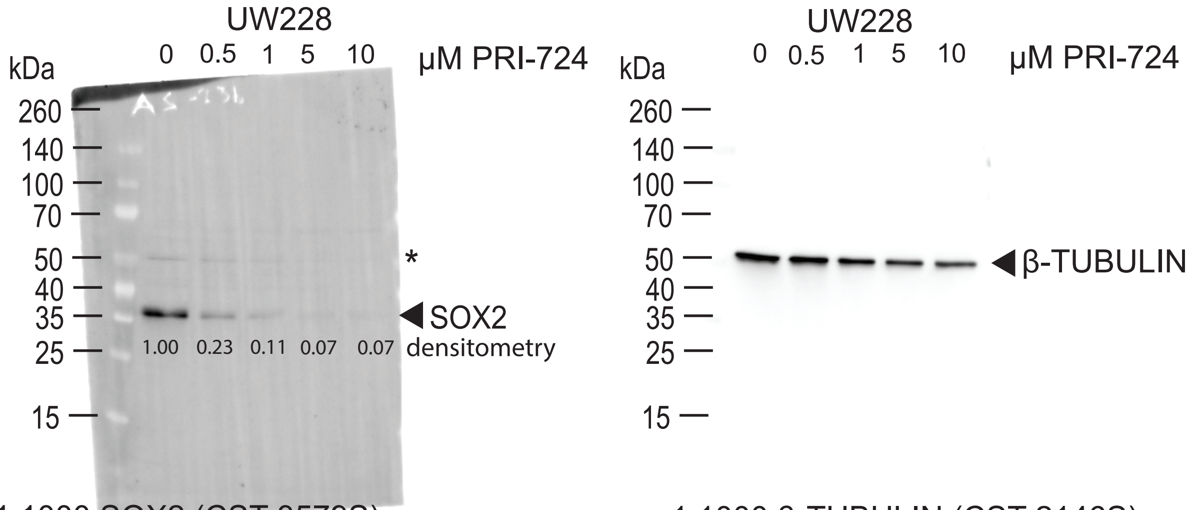

1:1000 SOX2 (CST 3579S)
1:5000 anti-rabbit secondary (CST 7054S)

1:1000 β-TUBULIN (CST 2146S)
1:5000 anti-rabbit secondary (CST 7054S)

*Appears to be a non-specific band. We were unable to rule out the possibility of a post-translational modification.

B

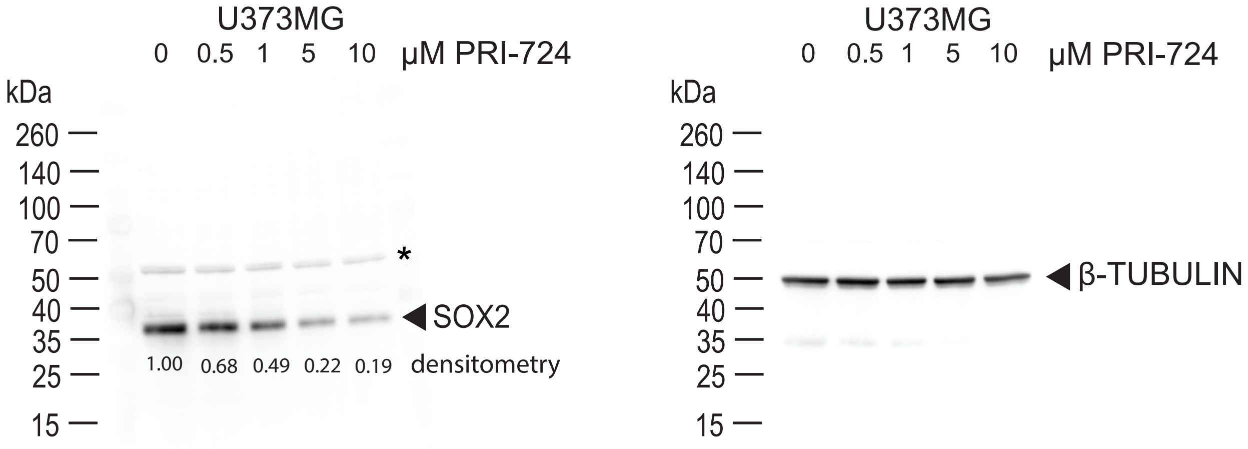

1:1000 SOX2 (CST 3579S)
1:5000 anti-rabbit secondary (CST 7054S)

1:1000 β-TUBULIN (CST 2146S)
1:5000 anti-rabbit secondary (CST 7054S)

*Appears to be a non-specific band. We were unable to rule out the possibility of a post-translational modification.

C

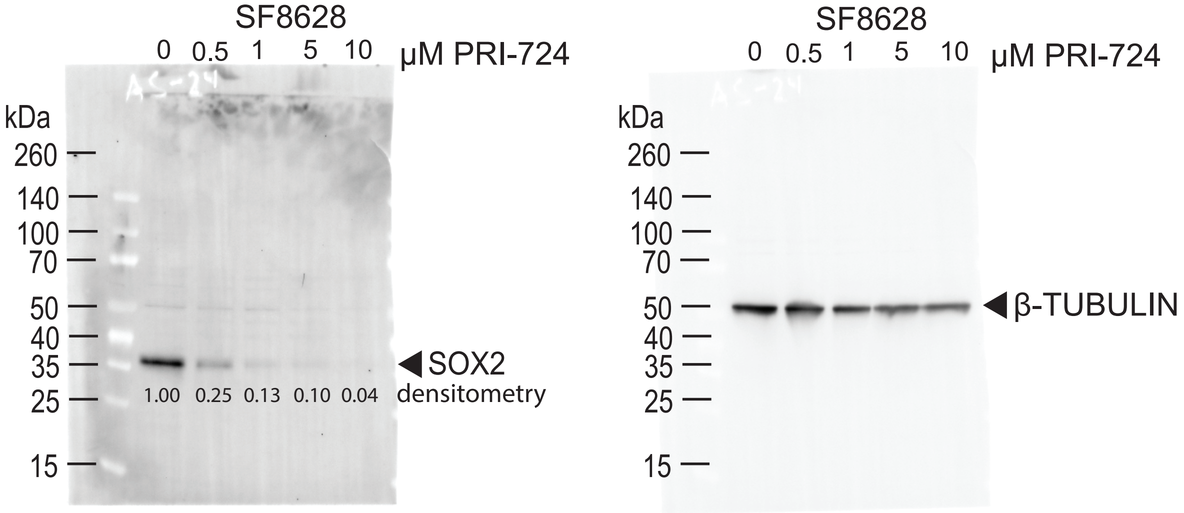

1:1000 SOX2 (CST 3579S)
1:5000 anti-rabbit secondary (CST 7054S)

1:1000 β-TUBULIN (CST 2146S)
1:5000 anti-rabbit secondary (CST 7054S)

Figure 2

D

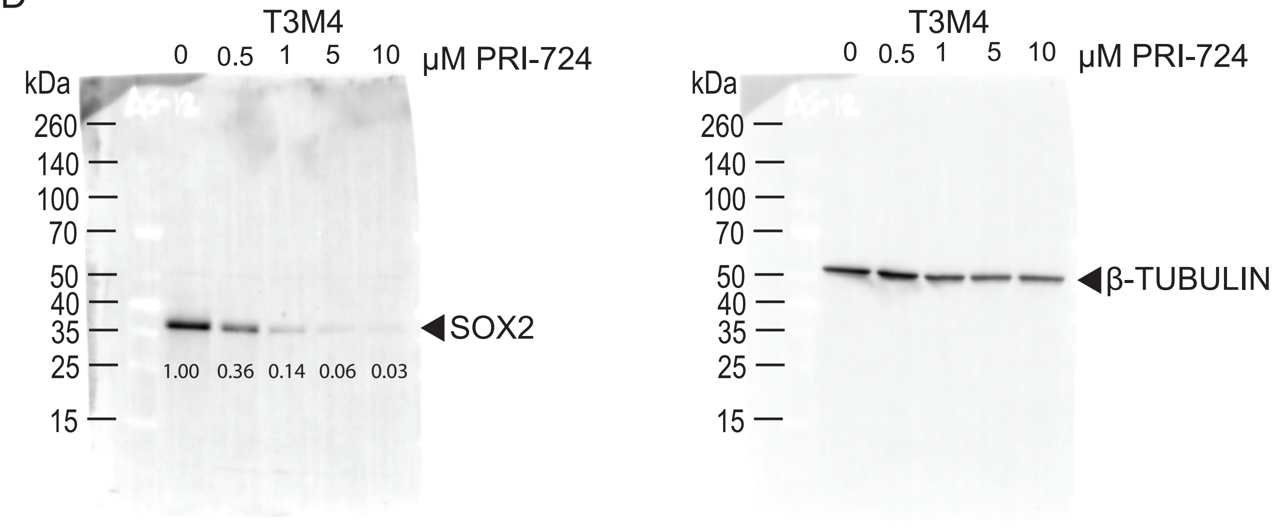

1:1000 SOX2 (CST 3579S)
1:5000 anti-rabbit secondary (CST 7054S)

1:1000 β-TUBULIN (CST 2146S)
1:5000 anti-rabbit secondary (CST 7054S)

E

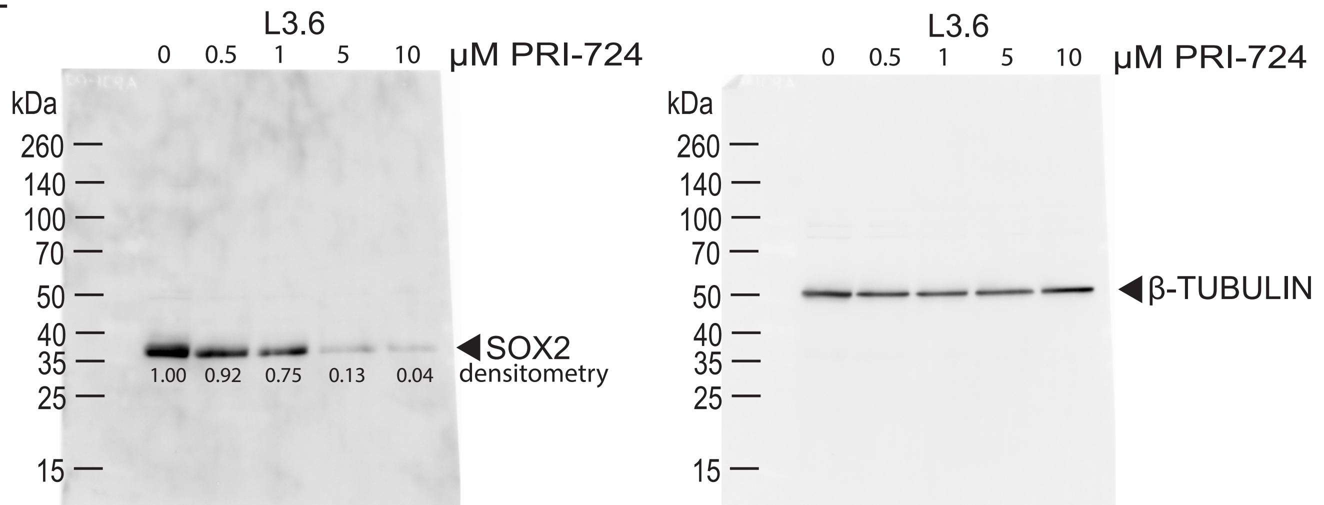

1:1000 SOX2 (CST 3579S)
1:5000 anti-rabbit secondary (CST 7054S)

1:1000 b-TUBULIN (CST 2146S)
1:5000 anti-rabbit secondary (CST 7054S)

F

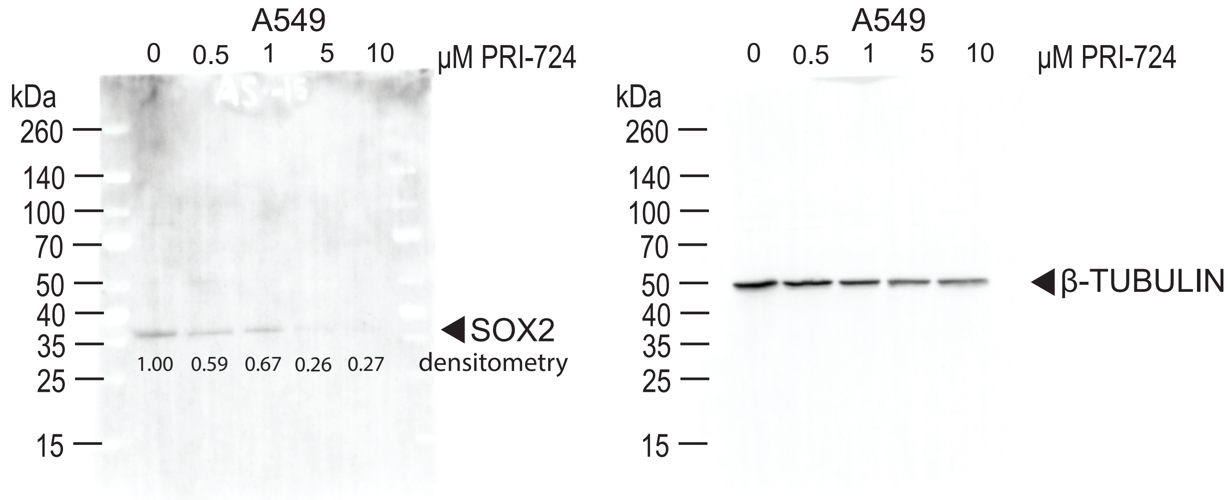

1:1000 SOX2 (CST 3579S)
1:5000 anti-rabbit secondary (CST 7054S)

1:1000 β-TUBULIN (CST 2146S)
1:5000 anti-rabbit secondary (CST 7054S)

Figure 2

G

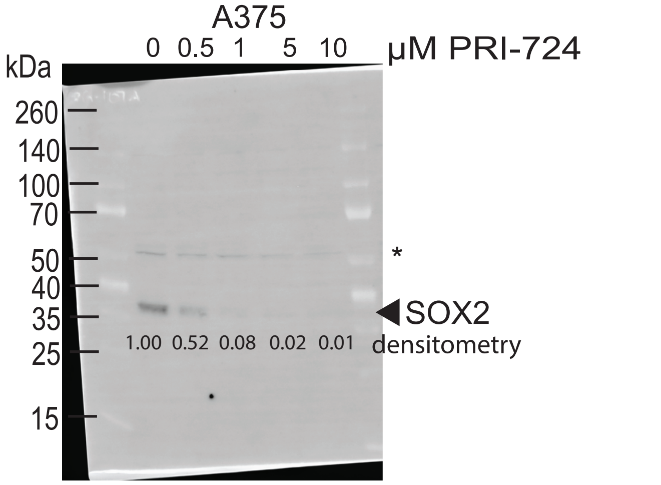

1:1000 SOX2 (CST 3579S)
1:5000 anti-rabbit secondary (CST 7054S)

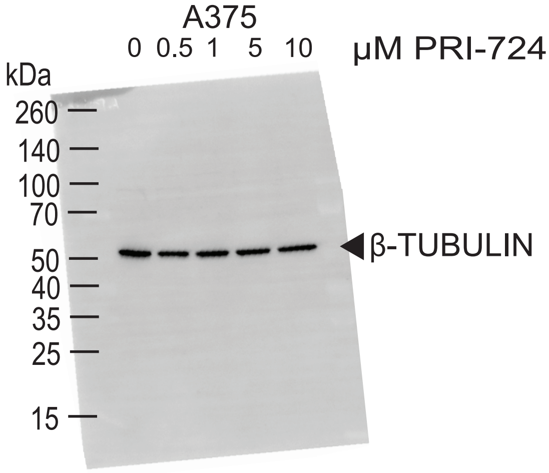

1:1000 β-TUBULIN (CST 2146S)
1:5000 anti-rabbit secondary (CST 7054S)

*Appears to be a non-specific band. We were unable to rule out the possibility of a post-translational modification.

H

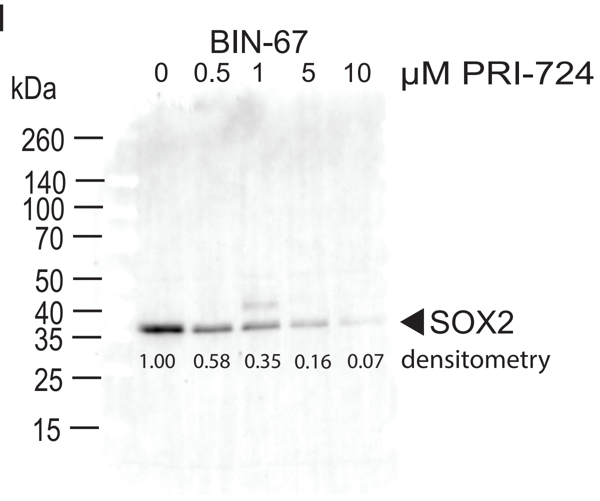

1:1000 SOX2 (CST 3579S)
1:5000 anti-rabbit secondary (CST 7054S)

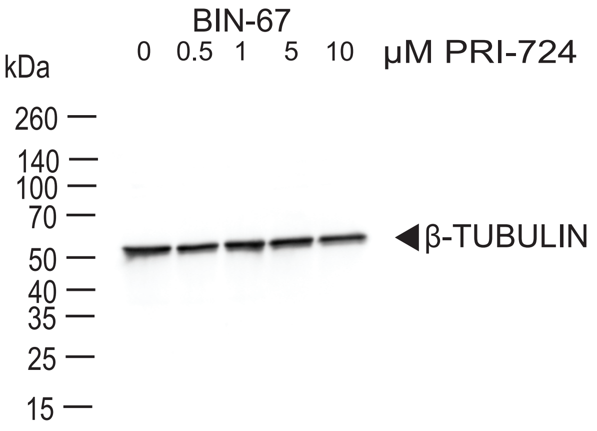

1:1000 β-TUBULIN (CST 2146S)
1:5000 anti-rabbit secondary (CST 7054S)

I

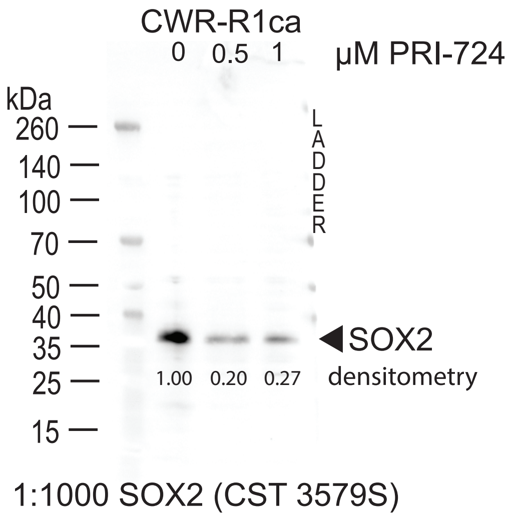

1:1000 SOX2 (CST 3579S)
1:5000 anti-rabbit secondary (CST 7054S)

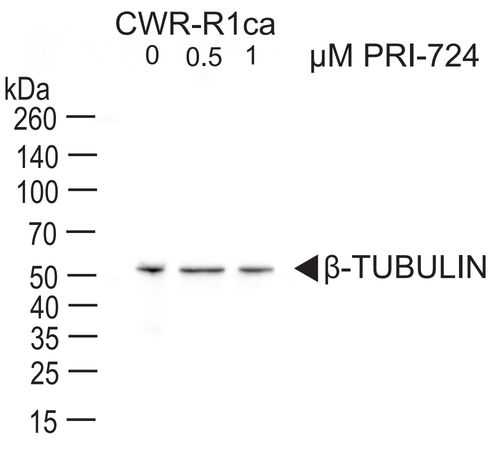

1:1000 β-TUBULIN (CST 2146S)
1:5000 anti-rabbit secondary (CST 7054S)

Figure 2 - Ran a new western with the same protein isolates, probed with an alternative SOX2 antibody.

A & G

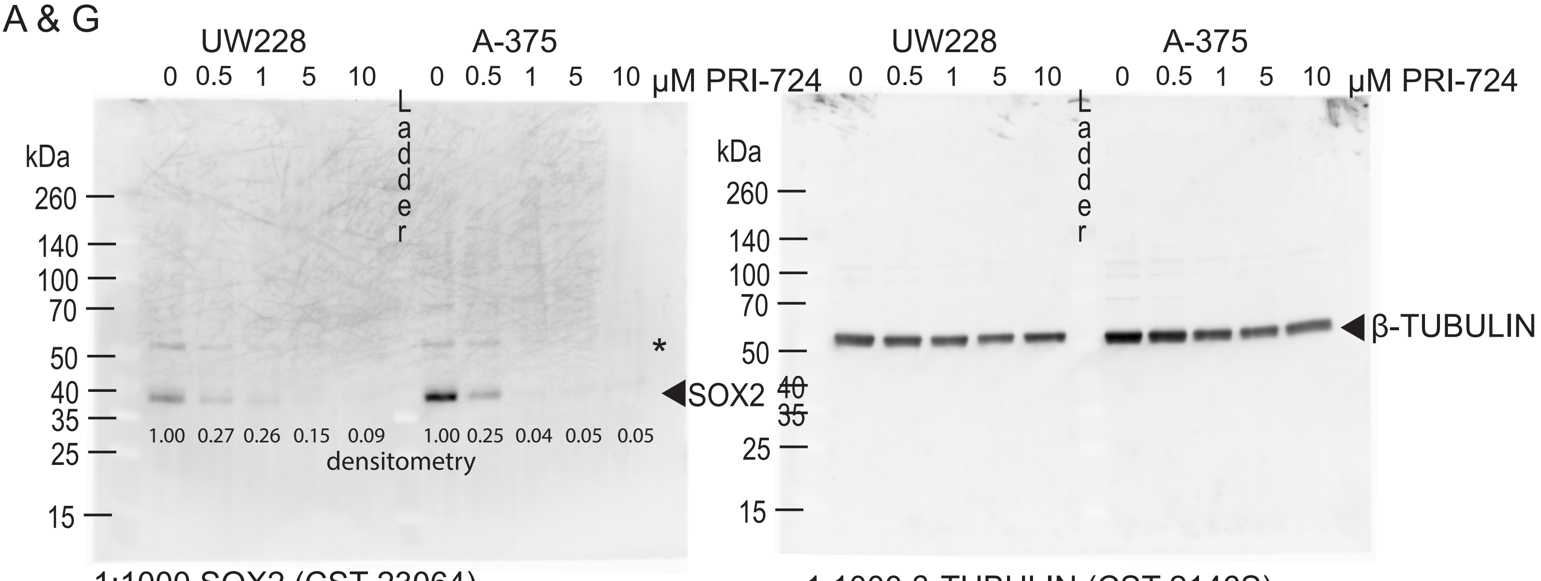

1:1000 SOX2 (CST 23064)
1:5000 anti-rabbit secondary (CST 7054S)

1:1000 β-TUBULIN (CST 2146S)
1:5000 anti-rabbit secondary (CST 7054S)

*Appears to be a non-specific band. We were unable to rule out the possibility of a post-translational modification.

B

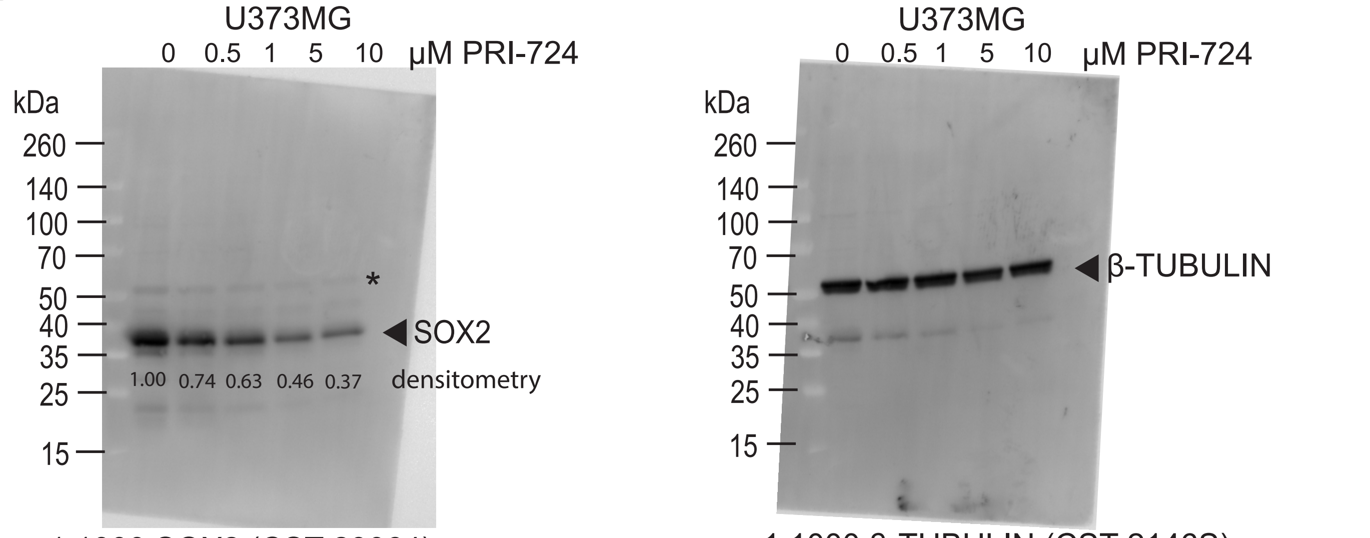

1:1000 SOX2 (CST 23064)
1:5000 anti-rabbit secondary (CST 7054S)

1:1000 β-TUBULIN (CST 2146S)
1:5000 anti-rabbit secondary (CST 7054S)

*Appears to be a non-specific band. We were unable to rule out the possibility of a post-translational modification.

Figure 3

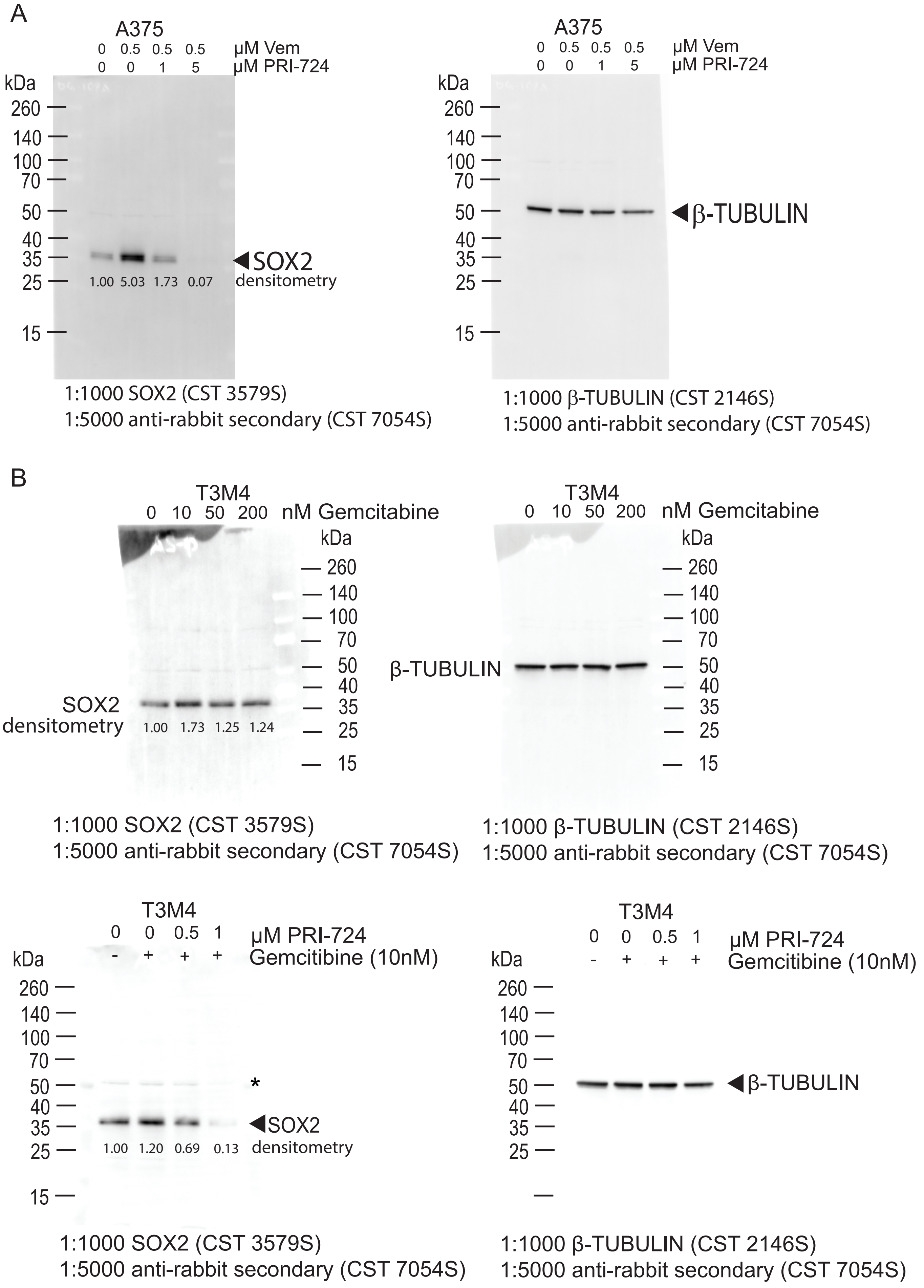

* Appears to be a non-specific band. We were unable to rule out the possibility of a post-translational modification.

Figure 3

C

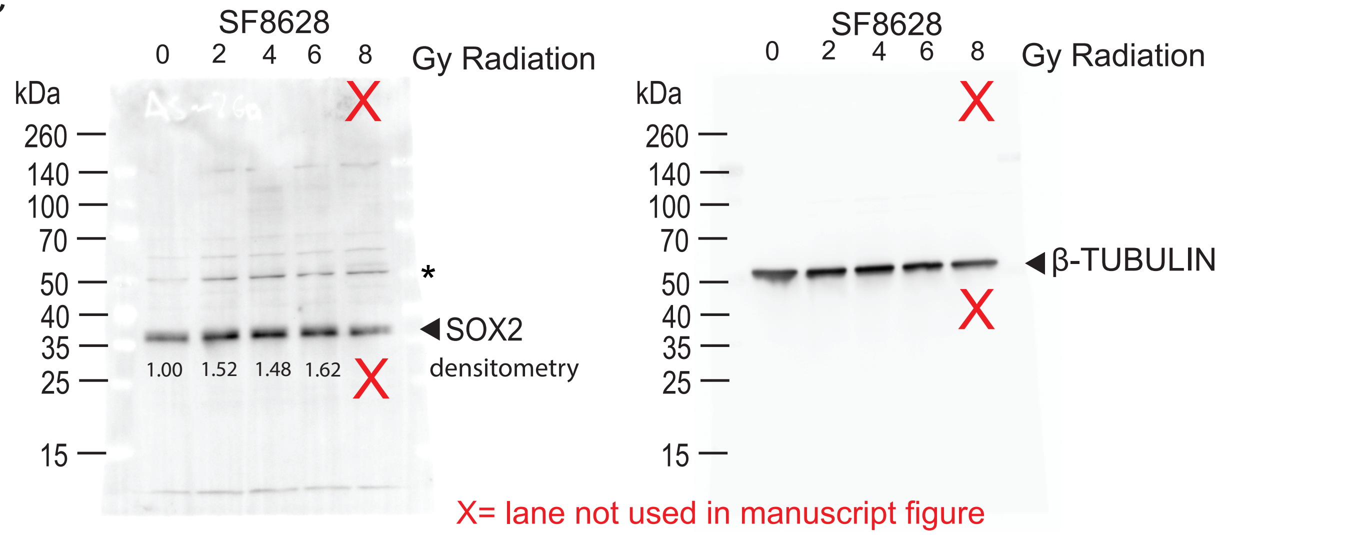

1:1000 SOX2 (CST 3579S)
1:5000 anti-rabbit secondary (CST 7054S)

1:1000 β-TUBULIN (CST 2146S)
1:5000 anti-rabbit secondary (CST 7054S)

*Appears to be a non-specific band. We were unable to rule out the possibility of a post-translational modification.

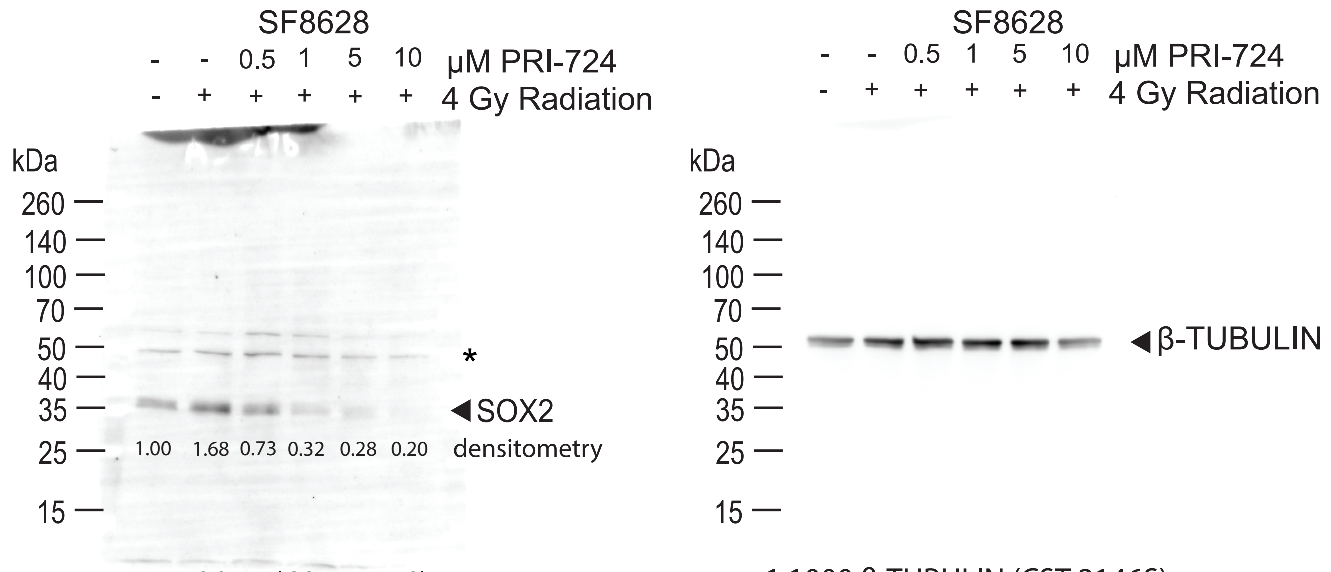

1:1000 SOX2 (CST 3579S)
1:5000 anti-rabbit secondary (CST 7054S)

1:1000 β-TUBULIN (CST 2146S)
1:5000 anti-rabbit secondary (CST 7054S)

*Appears to be a non-specific band. We were unable to rule out the possibility of a post-translational modification.

Figure 4

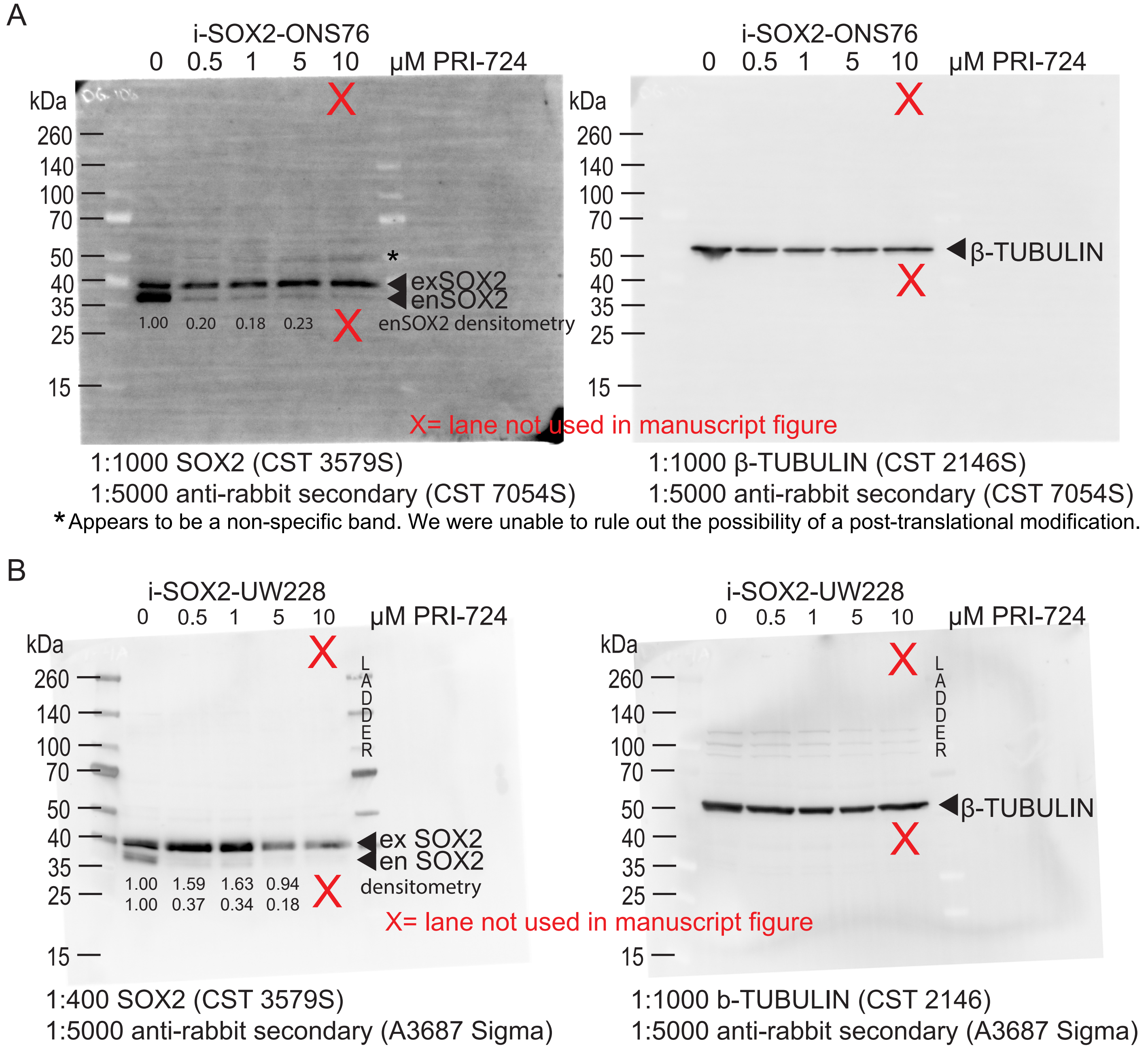
A
i-SOX2-ONS76
0 0.5 1 5 10 µM PRI-724
kDa
260
140
100
70
50
40
35
25
15
*
exSOX2
enSOX2
1.00 0.20 0.18 0.23
enSOX2 densitometry
X
X
i-SOX2-ONS76
0 0.5 1 5 10 µM PRI-724
kDa
260
140
100
70
50
40
35
25
15
β-TUBULIN
X
X
X= lane not used in manuscript figure
1:1000 SOX2 (CST 3579S)
1:5000 anti-rabbit secondary (CST 7054S)
1:1000 β-TUBULIN (CST 2146S)
1:5000 anti-rabbit secondary (CST 7054S)
*Appears to be a non-specific band. We were unable to rule out the possibility of a post-translational modification.
B
i-SOX2-UW228
0 0.5 1 5 10 µM PRI-724
kDa
260
140
100
70
50
40
35
25
15
LADDER
ex SOX2
en SOX2
1.00 1.59 1.63 0.94
1.00 0.37 0.34 0.18
densitometry
X
X
i-SOX2-UW228
0 0.5 1 5 10 µM PRI-724
kDa
260
140
100
70
50
40
35
25
15
LADDER
β-TUBULIN
X
X
X= lane not used in manuscript figure
1:400 SOX2 (CST 3579S)
1:5000 anti-rabbit secondary (A3687 Sigma)
1:1000 b-TUBULIN (CST 2146)
1:5000 anti-rabbit secondary (A3687 Sigma)

Figure 7

Run of ONS76 cells treated with 1μM PRI-724 for 48 hours then either sustained (S) treatment for another 72 hours or treatment was removed (R). Whole cell extracts (Half density 250000 cells or Whole density 500000 cells) Control (C) cells were treated with DMSO.

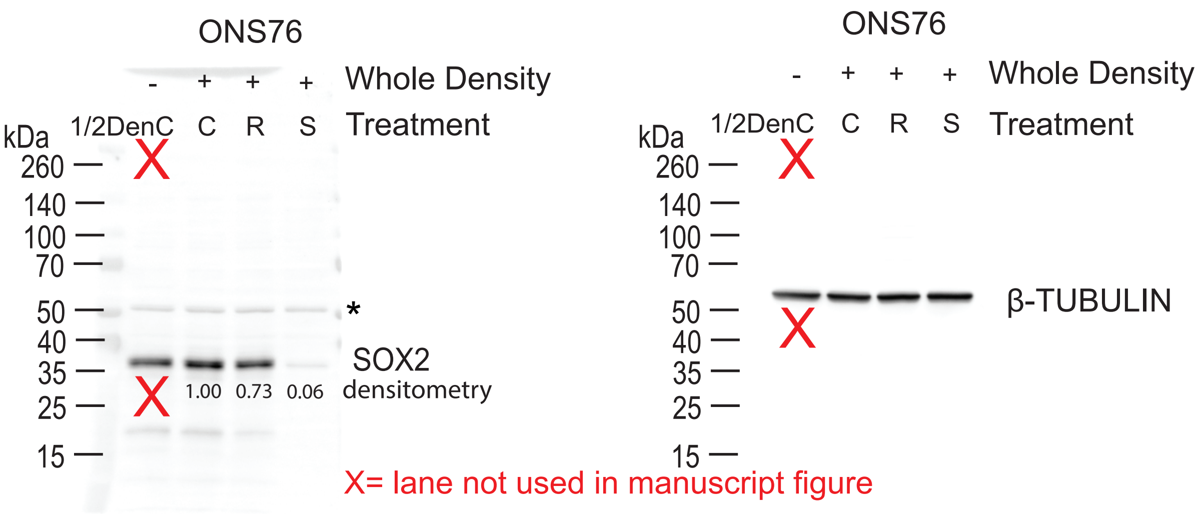

1:1000 SOX2 (CST 3579S)
1:5000 anti-rabbit secondary (CST 7054S)

1:1000 β-TUBULIN (CST 2146S)
1:5000 anti-rabbit secondary (CST 7054S)

*Appears to be a non-specific band. We were unable to rule out the possibility of a post-translational modification.
